# Novel molecular imaging ligands targeting matrix metalloproteinases 2 and 9 for imaging of unstable atherosclerotic plaques

**DOI:** 10.1371/journal.pone.0187767

**Published:** 2017-11-30

**Authors:** Nazanin Hakimzadeh, Victorine A. Pinas, Ger Molenaar, Vivian de Waard, Esther Lutgens, Berthe L. F. van Eck-Smit, Kora de Bruin, Jan J. Piek, Jos L. H. Eersels, Jan Booij, Hein J. Verberne, Albert D. Windhorst

**Affiliations:** 1 Department of Biomedical Engineering & Physics, Academic Medical Center, University of Amsterdam, Amsterdam, The Netherlands; 2 Department of Cardiology, Academic Medical Center, Amsterdam, The Netherlands; 3 Department of Radiology and Nuclear Medicine, Academic Medical Center, University of Amsterdam, Amsterdam, The Netherlands; 4 Department of Radiology & Nuclear Medicine, VU University Medical Center, Amsterdam, The Netherlands; 5 BV Cyclotron VU, Amsterdam, The Netherlands; 6 Department of Medical Biochemistry, Academic Medical Center, Amsterdam, The Netherlands; 7 Institute for Cardiovascular Prevention (IPEK) Ludwig Maximilian's University, Munich, Germany; National Center For Scientific Research Demokritos, GREECE

## Abstract

Molecular imaging of matrix metalloproteinases (MMPs) may allow detection of atherosclerotic lesions vulnerable to rupture. In this study, we develop a novel radiolabelled compound that can target gelatinase MMP subtypes (MMP2/9) with high selectivity and inhibitory potency. Inhibitory potencies of several halogenated analogues of MMP subtype-selective inhibitors (N-benzenesulfonyliminodiacetyl monohydroxamates and *N*-halophenoxy-benzenesulfonyl iminodiacetyl monohydroxamates) were in the nanomolar range for MMP2/9. The analogue with highest inhibitory potency and selectivity was radiolabelled with [^123^I], resulting in moderate radiochemical yield, and high radiochemical purity. Biodistribution studies in mice, revealed stabilization in blood 1 hour after intravenous bolus injection. Intravenous infusion of the radioligand and subsequent autoradiography of excised aortas showed tracer uptake in atheroprone mice. Distribution of the radioligand showed co-localization with MMP2/9 immunohistochemical staining. In conclusion, we have developed a novel selective radiolabeled MMP2/9 inhibitor, suitable for single photon emission computed tomography (SPECT) imaging that effectively targets atherosclerotic lesions in mice.

## Introduction

Traditional diagnosis of atherosclerotic disease severity focused on the percentage of stenosis detected by angiography. This simplistic view, that atherosclerosis is merely characterized by pathological lipid deposition within arteries, is not inclusive of the complexities involving active inflammation, a vast lipid core with a thin fibrous cap, expansive remodeling, intraplaque hemorrhage and intraplaque neovascularization of the vasa vasorum [1]. The latter are defined as microvessels sprouting from the adventitial layer via angiogenesis in response to hypoxia and inflammation. This network of immature thin-walled vessels, are prone to leakage due to their lack of endothelial gap junctions, and thereby serve a highly pathological role by allowing infiltration of inflammatory cells, including macrophages and their respective proteolytic enzymes, matrix metalloproteinases (MMP) [2]. Accumulation of MMP activity in inflamed plaques has been suggested to mediate expansive vascular remodeling, following dissolution of extracellular matrix content in the fibrous cap [3]. These characteristics collectively contribute to destabilization of atherosclerotic lesions and thereby lead to potentially fatal events due to spontaneous plaque rupture (i.e. unstable plaques). Therefore, more advanced diagnostic tools are required to detect unstable plaques. In this regard imaging of MMP activity is an accretive approach, that could provide critical insight on the extent of vascular instability and thereby allow targeted treatment and evaluation of therapeutic intervention efficacy.

Other MMP molecular imaging tracers have been shown to target a large spectrum of MMPs including gelatinases, interstitial collagenases as well as broad specificity stromelysins [4, 5]. Collectively, the substrate repertoire of MMPs includes extracellular matrix (ECM) components including fibrillary collagens, elastin, matrix proteoglycan core proteins, in addition to non-matrix substrates [6]. The involvement of MMPs in ECM destabilization in atherosclerotic plaques consequently leads to plaque rupture, and can thereby lead to myocardial and cerebral infarction. Gelatinases (MMP2 and MMP9) have been shown to be the predominant MMPs secreted by T lymphocytes and macrophages [7, 8]. In the setting of atherosclerotic plaque development, T lymphocytes and macrophages occupy plaques throughout all stages and play a crucial role in modulating acute and chronic inflammatory responses [9, 10]. Smooth muscle cells, fibroblasts and endothelial cells stimulated by cytokines released by pro-inflammatory cells, are also large producers of MMP2/9.

Molecular-based single photon emission computed tomography (SPECT) and positron emission tomography (PET) imaging modalities have been used to evaluate key molecular processes involved in cardiovascular disease, including atherosclerosis, ventricular remodeling post myocardial infarction and ischemia-induced angiogenesis [11]. Relative to magnetic resonance imaging (MRI), nuclear imaging approaches are particularly well suited for *in vivo* molecular imaging due to their high sensitivity, acceptable spatial resolution as well as wide availability of instrumentation and molecular probes [12].

In this study we sought to develop a novel highly selective MMP radioligand suitable for molecular imaging of atherosclerotic lesions. To do this, we have examined the influence of halogen substitution on the change in affinity of previously described MMP inhibitors [13–16]. Halogen substituted analogs (fluorine, bromine and iodine) would ultimately deem the compounds suitable for radiolabeling and would thereby allow us to generate novel molecular imaging agents targeting MMPs. We have focused on sulfonamide based hydroxamic acids for halogenation, whereby the non-halogenated form of these compounds have previously been shown to be potent MMP inhibitors. Hydroxamate based MMP inhibitors are the strongest class of MMP inhibitors as they achieve bidentate binding to the Zinc ion region of MMPs, resulting in a distorted geometry. In a key publication of Santos et al. [14], novel non-peptidic hydroxamate-based MMP inhibitors capable of targeting the deep S1’ pocket of MMPs were introduced. We have thereby synthesized a series of halogenated sulfonamide based compounds based on the lead inhibitor presented by Santos et al. [14] as well as traditional sulfonamide based MMP inhibitors.

The inhibitory potency of these halogenated ligands towards MMP2 and MMP9 was determined, followed by radiolabeling of the iodine substituted ligand. Radiolabeling was conducted with [^123^I], as this is a suitable radionuclide for SPECT imaging. Finally, we examined the biodistribution of the selected radiolabeled ligand as well as conducted validation studies to confirm the efficacy of the tracer to selectively bind atherosclerotic lesions. Validation studies were conducted using *ex vivo* autoradiography and immunochemistry in an ApoE-/- mouse model, known for the spontaneous development of unstable atherosclerotic plaques, comprised of MMP2 and 9.

## Methods

Expanded methods section provided in S1 Appendix. ARRIVE Guidelines checklist reporting in vivo experiments provided in S2 Appendix.

### Chemical synthesis

All non-aqueous reactions were carried out under nitrogen atmosphere. Reagents and solvents were obtained from commercial sources and were used without further purification. Yields refer to purified products and are not optimized. Analytical thin layer chromatography (TLC) was performed on Merck silica gel 60 F254 aluminum-backed plates. Compounds were visualized by ultraviolet (UV) light (254 nm). Flash column chromatography was performed with kiesel gel 60 F_254_ (Baker). Nuclear Magnetic Resonance (NMR) spectra were recorded a Bruker Advance 400 MHz spectrometer (400 MHz for ^1^H, 100 MHz for ^13^C). Chemical shifts (δ) are expressed in parts per million (ppm) with the solvent signal as reference, 2.50 for DMSO-*d6*, 7.27 for CDCl_3_. Splitting patterns are designated as s (singlet), d (doublet), t (triplet), q (quartet), m (multiplet), br s (broad singlet). LC-MS (SHIMAZU, LCMS-2020 combined with LC(UFLC) UFLCXR) analyses were performed on either Acquity C-18 column (2.1 mm x 100 mm, 1.7 μm) using electrospray ionization (ESI) or on a Gemini C-18 column (4.6 mm x 50 mm, 5 μm) using atmospheric pressure chemical ionization (APCI). The purity of all tested compounds was determined by liquid chromatography-mass spectrometry (LC-MS) and elemental analyses, resulting in a purity < 96% for all tested compounds. All commercially obtained chemicals were used without prior purification.

### In vitro MMP inhibition & selective binding capacity assays

The inhibitory potency and selective binding of MMP-ligands was investigated by examining their capacity to block MMP1, MMP2 and MMP9 activity, using *in vitro* fluorometric assays (SensoLyte^TM^ 520 MMP-1, SensoLyte^TM^ 520 MMP-2, SensoLyte^TM^ 520 MMP-9; AnaSpec, San Jose, USA). Assay buffer was warmed to 37°C and used to dilute the reference inhibitor N-isobutyl-N-(4-methoxyphenylsulfonyl)glycyl hydoxamic acid (NNGH 1:100), calibration standard (1:50), substrate and enzyme (1:60). Components were loaded into a 96-well plate. MMP ligands **10a-d** were also aliquoted to the respective 96-well plate at varying concentrations (10pM, 100pM, 1nM, 10nM, 100nM, 1μM, 10 μM and 100 μM), followed by incubation at 37°C for 30 minutes. Reactions were initiated by addition of the substrate to the respective wells, after which fluorescence generated was measured using a microplate reader (Synergy HT Multi-detection Microplate Reader, BioTek, 310–405nM) continuously for 30 minutes at 60 second intervals. Data analysis was conducted using Sigma Plot and Microsoft Excel.

### In vivo biodistribution

All experiments were approved by the institutional committee for animal experiments at the Academic Medical Center. C57BL/6 female mice (14–16 week) were purchased from Harlan and housed in the animal care facility at the Academic Medical Center. Animals were anesthetized using 126 mg ketamine, 100 μg dexdomitor, 500 μg atropine, 7.5 mL NaCl 0.9% of which 0.015 mL/g body weight was administered by intraperitoneal injection. In order to investigate the *in vivo* biodistribution of MMP ligand [^123^I]**10c**, 4 MBq was administered intravenously in the tail vein and allowed to circulate for either 5, 15, 30, 60 minutes or 2, 3, 4, 6 and 24 hours (n = 3–4 animals for each time point). At the end of each circulation time, animals were sacrificed by bleeding through phosphate buffered saline solution (PBS) perfusion. After the animals were sacrificed, whole blood was collected by cardiac puncture, and selected organs were excised (heart, lungs, liver and kidneys). The organs were weighed and radioactivity was measured using a scintillation counter (TRT-CARB 2000CA, Perkin Elmer 1987). The measured radioactivity was expressed as the percentage of the injected dose per gram of tissue per gram of body weight (% ID/g/g).

### In vivo binding to atherosclerotic plaques

To investigate selective binding of [^123^I]**10c** to MMP2/9 found in atherosclerotic lesions, we examined the distribution of the [^123^I]**10c** in 8–10 week old female ApoE-/- mice (n = 5, Charles River) that were fed a high fat Western diet (15% fat, 0.25% cholesterol; Arie Blok Diervoeding, Netherlands) for 4 weeks. These animals subsequently developed atherosclerotic plaques. 40 MBq of [^123^I]**10c** were administered by intravenous injection in the tail vein and allowed to circulate for 1 hour. Animals were euthanized by bleeding via PBS infusion. The aortic arch was excised and 10 μm serial sections were made. Aortic arch sections were placed on a phosphor imaging plate (Fuji) and the amount of radioactivity was measured using a storage phosphor imaging apparatus (Typhoon Trio; GE Healthcare Life Sciences). Sections were then stained with hematoxylin/eosin staining, or used for MMP2 and MMP9 detection. Prior to incubation with monoclonal goat-anti-mouse MMP2 (R&D Systems) and rabbit-anti-mouse MMP9 (Abcam), the sections were heated to improve antigen retrieval. Rabbit-anti-goat secondary antibodies (DAKO) were utilized on the MMP2 sections. Thereafter, all sections were probed with the PowerVision anti-rabbit polymer, conjugated to horseradish peroxidase (Immunologic), to reveal positive primary antibody binding by means of chromogen conversion of 3,3’-diaminobenzidine (DAB), followed by nuclear counterstaining with hematoxylin. Histological sections were imaged with a bright-field microscope. The respective section used for MMP9 staining was also subsequently stained for smooth muscle cells. After the MMP9 staining, the section was incubated with anti-alpha-smooth muscle actin antibody (clone 1A4; DAKO) in Tris buffered saline (TBS) with 1% normal goat serum for 1h at room temperature. Thereafter, the section was washed in TBS and incubated with BrightVision poly alkaline phosphatase (AP)-anti Mouse IgG (ImmunoLogic) for 30 min, followed by TBS washing and incubation with Vector Blue AP substrate (SK-5300; Vector Laboratories Inc) for 1h. Similar to the other histological sections, imaging was conducted with a bright-field microscope.

## Results

**Fig 1:**
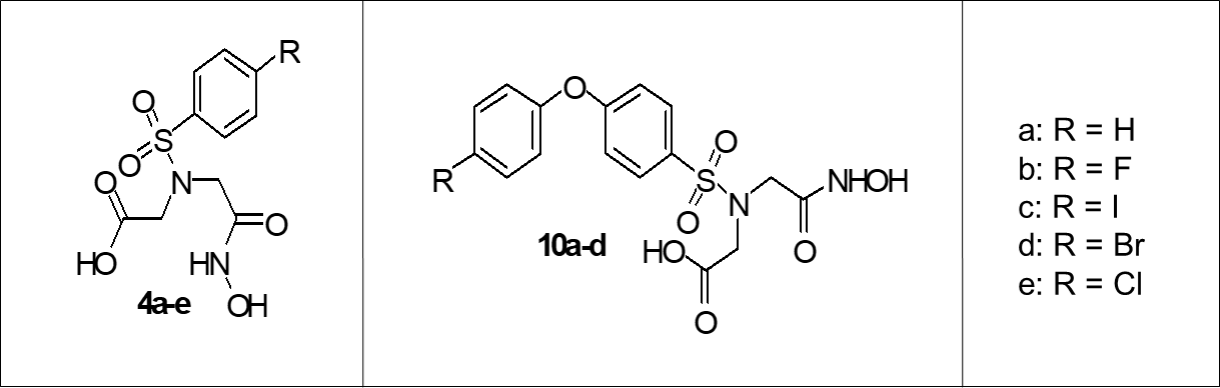
Hydroxamate target compounds. Schematic depicting halogenated forms of two sulfonamide hydroxamate based compounds examined (**4a-e** and **10a-d**).

We synthesized and examined two series of halogenated sulfonamide hydroxamate based compounds for MMP inhibition, **4a-e** and **10a-d** (Fig 1, Table 1). Our goal was to investigate the effects of halogenation on these compounds such that they could be ultimately utilized for radiosynthesis.

**Table 1:** List of halogenated forms of two sulfonamide hydroxamate based compounds examined.

Compound Symbol	Compound name
**4a**	*2-(N-(2(hydroxyamino)-2-oxoethyl)phenylsulfonamido) acetic acid*
**4b**	*2-(4-fluoro-N-(2-hydroxyamino)-2oxoethyl)phenylsufonamido acetic acid*
**4c**	*2-(4-Iodo-N-(2-hydroxyamino)-2-oxoethyl) phenylsufonamido acetic acid*
**4d**	*2-(4-bromo-N-(2-hydroxyamino)-2-oxoethyl) phenylsufonamido acetic acid*
**4e**	*2-(4-chloro-N-(2-hydroxyamino)-2-oxoethyl)phenylsufonamido acetic acid*
**10a**	*([4-Phenoxy-benzenesulphonyl]-hydroxycarbamoylmethyl-amino) acetic acid*
**10b**	*[4-(4-Fluoro-phenoxy)-benzenesulphonyl]-hydroxycarbamoylmethyl-amino) acetic acid*
**10c**	*([4-(4-Iodo-phenoxy)-benzenesulphonyl]-hydroxycarbamoylmethyl-amino) acetic acid*
**10d**	*([4-(4-Bromo-phenoxy)-benzenesulphonyl]-hydroxycarbamoylmethyl-amino) acetic acid*

### Chemistry

**Fig 2:**
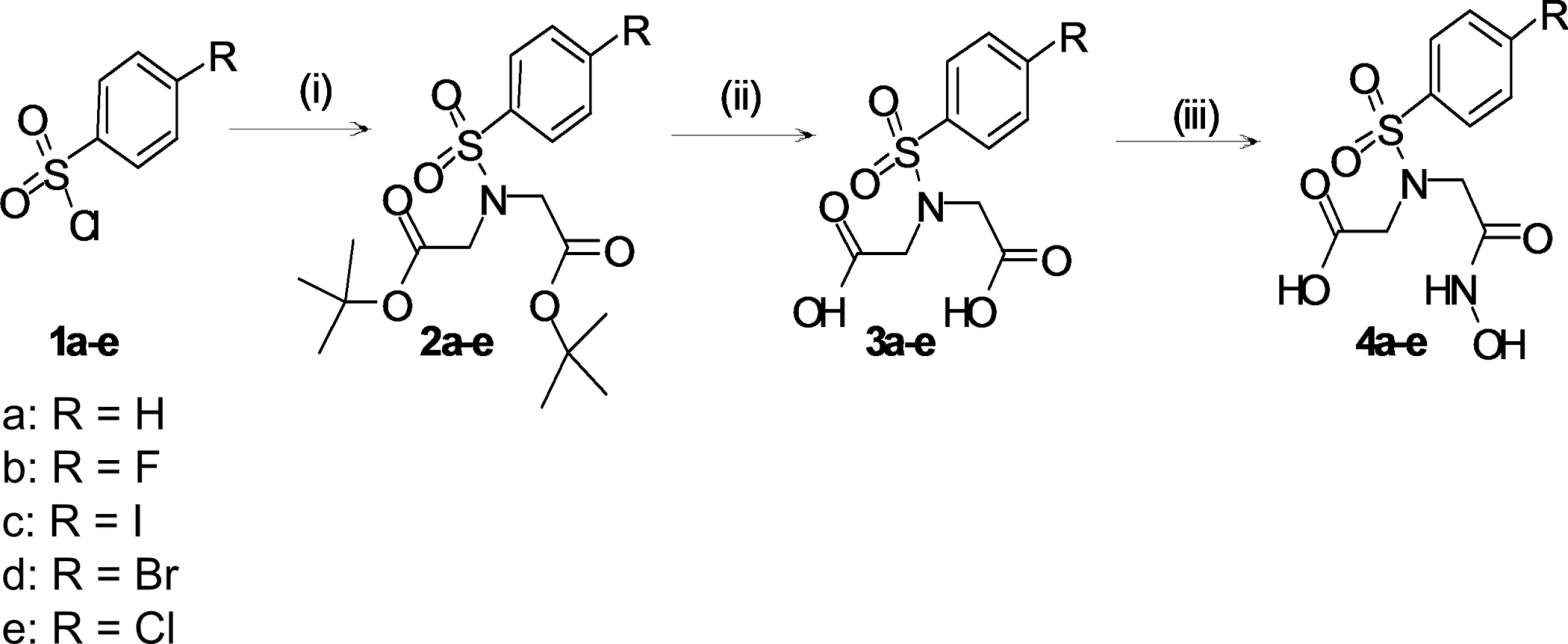
Synthesis of hydroxamate target compound 4a-e from 1a-e. (i): HN(CH_2_CO_2_*t*bu)_2_,TEA, DCM, rt, o/n, yield 39–75%; (ii): HCOOH, rt, o/n, yield 30–93%; (iii): ECF, NMM, THF, NH_2_OH x HCl, MeOH, 0°C, 2 h, yield 63–97%.

The commercially available compounds **1a-e** were converted into the intermediate tertiary butyl protected iminodiacetic acid derivatives **2a-e** in the presence of triethylamine in dichloromethane by reaction with di-tert-butyl iminodiacetate (Fig 2). Deprotection was achieved by stirring **2a-e** overnight in formic acid at room temperature to give the dicarboxylic acid compounds **3a-e**. After selective activation of one carboxylic acid group of the *N*-substituted iminodiacetic acid derivatives with ethylchloroformate in the presence of *N*-methylmorpholine and subsequent reaction with hydroxylamine in dichloromethane the target compounds **4a-e** were obtained in moderate overall yields.

**Fig 3:**
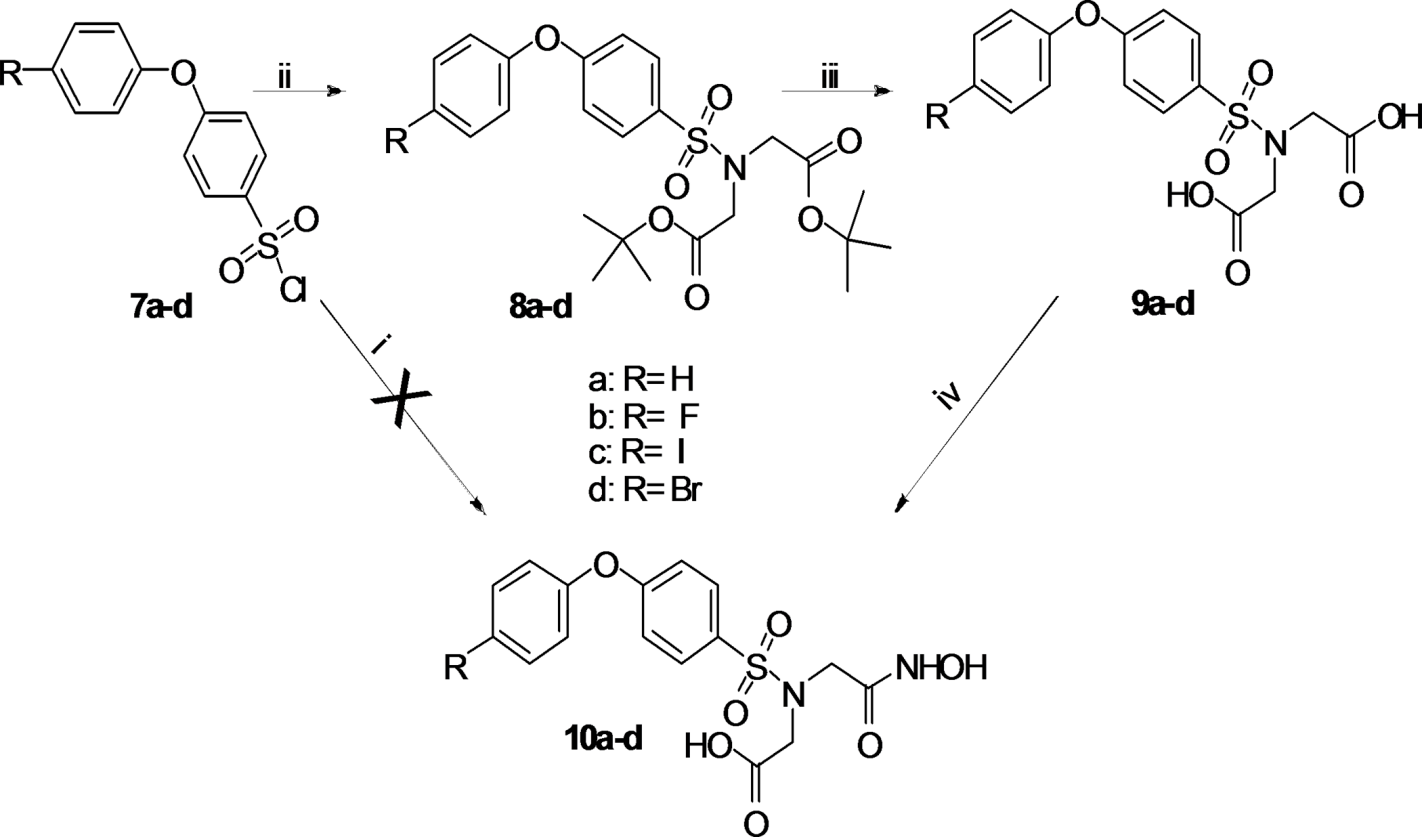
**Synthesis of 10 a-d.** (i): HN(CH_2_COOH, THF/H_2_O, rt, o/n, yield 3–35%; (ii): HN(CH_2_CO_2_*t*bu)_2_, TEA, DCM, rt, o/n, 78–99%; (iii): HCOOH, rt, o/n, yield 75–94%; (iv): ECF, NMM, THF, NH_2_OH x HCl, MeOH, 0°C, 2 h, yield 52–91%.

The arylsulfonyl chlorides **7a-d** were prepared from the commercially available compounds **5a-d**, introduction of the sulphonic acid moiety was achieved with chlorosulphonic acid leading to **6a-d** which were treated without intermediate purification with thionyl chloride to yield **7a-d** (S1 Fig). Subsequent synthesis of **9a-d** in a heterogeneous THF/H_2_O system (Schotten-Baumann conditions) as described previously [14] was unsuccessful. Alternatively, reaction of di-*tert*-butyl protected iminodiacetic acid with **7a-d** in the presence of triethylamine, followed by deprotection with formic acid yielded **9a-d**. Selective activation of one carboxylic acid group and condensation was performed using the same strategy as described for compounds **4a-e** and provided the target compounds **10a-d** (Fig 3). The purity of all tested compounds was found to be > 96% and was determined with liquid chromatography-mass spectrometry and elemental analysis.

### Inhibitory potency of candidate ligands towards MMP2 and 9

To determine the capacity of each candidate ligand to inhibit the activity of MMP2 and MMP9, as well as to examine their selectivity for these MMP subtypes, *in vitro* inhibitory potency assays were conducted with the compounds **4a-e** and **10a-d**. As depicted in Table 2, all compounds demonstrated inhibitory potency in the nanomolar range. Relative to compounds **4a-e**, ligands **10a-d** showed higher inhibitory potency towards MMP2/9, with less binding towards MMP1, and thus showed the desired selectivity.

**Table 2:** MMP inhibitory potencies of compounds 4a-e and 10a-d. IC_50_: half maximal inhibitory concentration. Values expressed in nM are presented as mean ± SD of three independent experiments in duplicate. Selectivity was calculated as the ratio of IC_50_ values; MMP1 IC_50_ for each respective compound was divided by the respective MMP2 or MMP9 IC_50_. The higher the selectivity value, the greater the selectivity towards either MMP2 or MMP9 relative to MMP1 for each respective compound.

	IC_50_ (nM)			Selectivity	
Compound	MMP1	MMP2	MMP9	MMP1/MMP2	MMP1/MMP9
**4a**	51 ± 0.10	39 ± 0.16	14 ± 1.05	1.31	3.64
**4b**	75 ± 0.15	12 ± 0.17	21 ± 0.19	6.25	3.57
**4c**	59 ± 0.17	27 ± 1.00	11 ± 1.14	2.19	5.36
**4d**	63 ± 0.13	19 ± 0.19	23 ± 0.21	3.32	2.74
**4e**	61 ± 0.11	41 ± 0.19	18 ±0.16	1.49	3.39
**10a**	1670 ± 980	1.89 ± 1.11	4.31 ± 2.06	884	387
**10b**	4690 ± 2220	0.13 ± 0.03	0.27 ± 0.15	3.60 x 10^4^	1.74 x 10^4^
**10c**	910 ± 560	0.87 ± 0.40	1.95 ±1.10	1.05 x 10^3^	467
**10d**	1320 ± 790	7.64 ± 1.43	1.61 ±1.41	173	820

### Radiosynthesis of ^123^I-N-arylsulfonyl iminodiacetyl monohydroxamate ([^123^I]10c)

Radiolabelling was considered only for compounds **10a-d**. Furthermore, although compound **10b** showed the highest affinity for MMP2/9 and selectivity towards MMP1, compound **10c** was selected as the most suitable candidate for targeting MMP2/9 since its affinity for MMP2/9 was high as well was its selectivity. More importantly, the chemical structure offers the opportunity for radiolabeling with Iodine-123. This radioisotope is suitable for SPECT imaging which could in turn be used for SPECT imaging in small animals. In addition, the relatively long half-life of Iodine-123 (13.2 hours) infers that a dedicated on-site radionuclide production facility is not required for utilization of compounds labelled with this radionuclide. Finally, SPECT scanners are more readily available than PET scanners.

Several routes for the radiosynthesis of ^123^I-*N*-arylsulfonyl iminodiacetyl monohydroxamate [^123^I]**10c** were explored. In a first attempt electrophilic iodination was investigated with the stannylated precursor **11**, which was synthesized starting from **10c** (S2 Fig). Unfortunately only starting material was recovered after the radiolabeling and no radiolabeled product was obtained. Protection of the hydroxymate function with a *t*-butyl moiety also did not generate radiolabeled product. The labeling of the stannylated di-*t*-butyl protected analogue of **8c** (S3 Fig) was however successful, [^123^I]**8c** was obtained in > 90% radiochemical yield. Compound [^**123**^I]**8c** was subsequently deprotected under acidic conditions into [^123^I]**9c.** The target compound, [^123^I]**10c** was finally obtained from [^123^I]**9c** after evaporation of the acidic reagents, by hydroxylamine condensation in basic conditions with a radiochemical purity of > 98% (S3 Fig). Drawback of this 3-step radiosynthesis route, besides the non-favorable multistep reaction sequence with radiolabeled compounds, was the deprotection step to form [^123^I]**9c** using 2 M hydrochloric acid in diethylether. This reaction gave rise to several side products, resulting is a low overall radiochemical yield of 4 ± 1% (corrected for decay).

**Fig 4:**
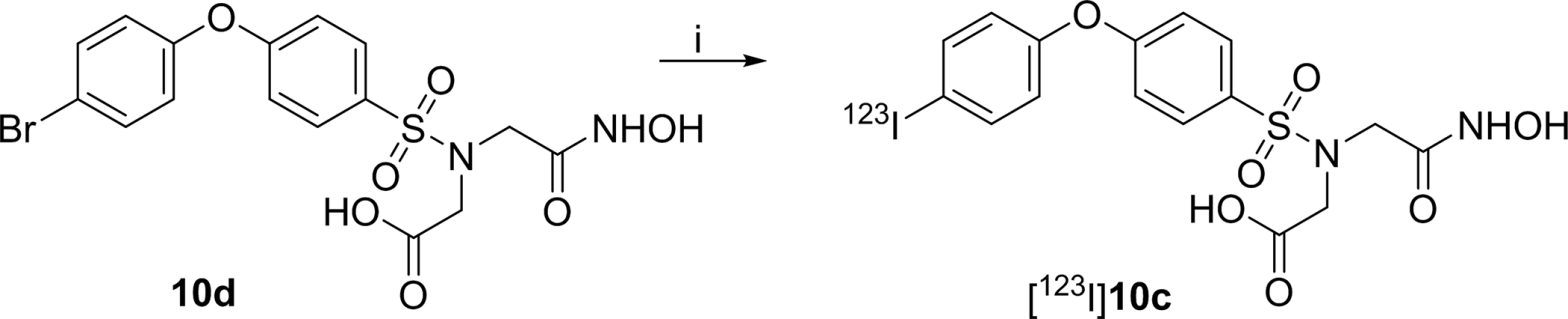
Radiosynthesis of [^123^I]10c from 10d by nucleophilic radioiodination. (i): Na[^123^I]I, ethanol, citric acid, 2,5 dihydroxybenzoic acid, CuSO_4_, SnSO_4,_ 130°C, 40 min, yield 51%.

Since this radiolabeling procedure was complicated and the yield not adequate to perform experiments with the radiolabeled product, the radiolabeling starting from the bromine precursor via a Cu^+^ assisted nucleophilic aromatic substitution was also investigated (Fig 4). This approach is advantageous since it can be performed in one reaction step in contrast to the complex multistep procedure. The elevated reaction temperature however, is a serious disadvantage since the risk for the formation of side products under these reaction conditions is increased. In addition, performing a nucleophilic aromatic substitution reaction is technically more complicated compared to a electrophilic reaction. Nonetheless, by means of nucleophilic aromatic substitution reaction conditions, radiolabeling of the target compound [^123^I]**10c**, resulted the desired product in a decay-corrected radiochemical yield and purity 38–63% and > 98%, respectively.

### In vivo biodistribution of [^123^I]10c

**Fig 5:**
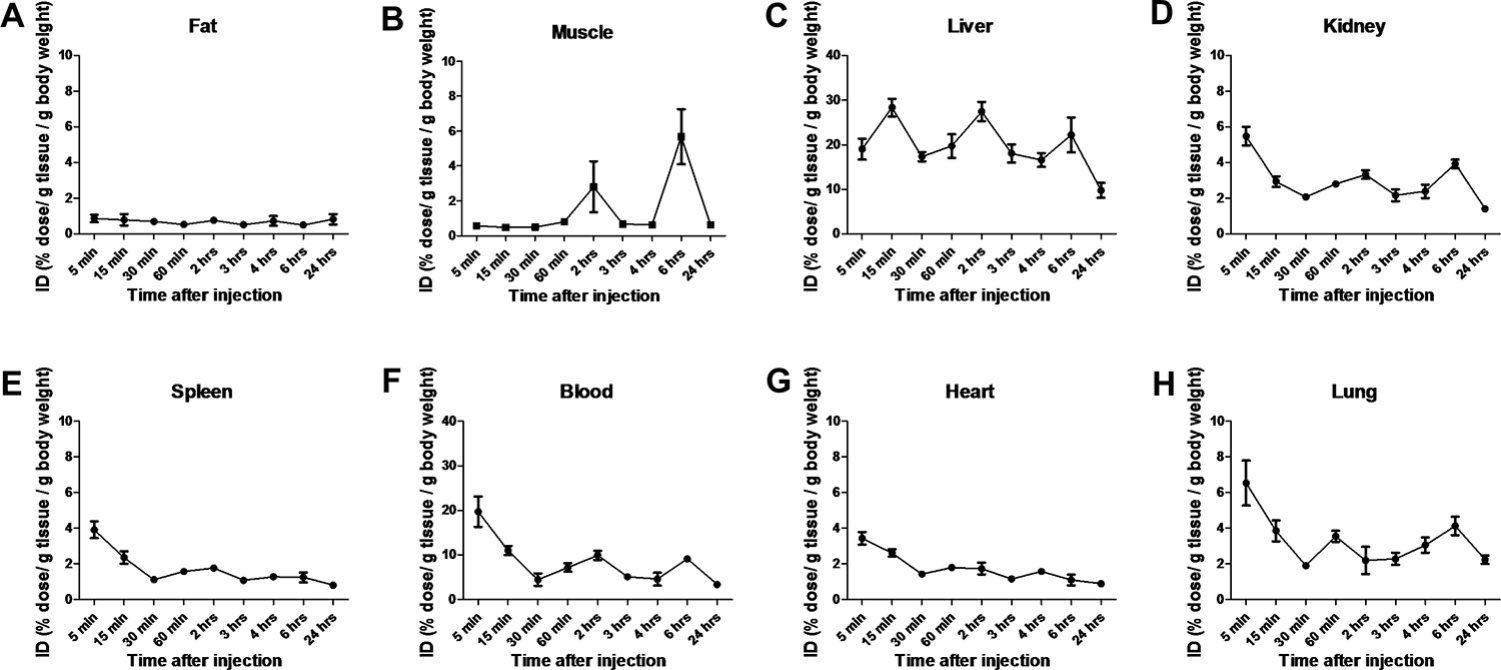
Biodistribution of [^123^I]10c following intravenous infusion in healthy mice (n = 3–4 /group). Injected dose (ID) of radioactivity was measured in fat (A), muscle (B), liver (C), kidney (D), spleen (E), blood (F), heart (G) and lung (H) at varying time-points after injection. Values shown are mean ± SEM.

The biodistribution of [^123^I]**10c** was investigated following intravenous infusion of 4 MBq in healthy C57BL/6 mice. Following the bolus injection, the compound was allowed to circulate for 5, 15, 30, 60 minutes, 2, 3, 4, 6 and 24 hours. As shown in Fig 5, radioactivity was highest in the liver and blood, while accumulation in fat was the lowest. In the blood, stabilization of the compound was reached after at least 1 hour of circulation.

### Uptake of [^123^I]10c in atherosclerotic lesions

To validate the efficacy of [^123^I]**10c** as an appropriate tracer for detection of atherosclerotic plaques, we examined the *in vivo* uptake of this ligand in ApoE-/- mice that were fed a high-fat diet. ApoE-/- mice develop atherosclerotic lesions spontaneously due to lack of ApoE expression, while the 6 week high fat diet accelerates lesion formation [17]. 40 MBq of [^123^I]**10c** was administered intravenously and allowed to circulate for 3 hours, after which the animals were euthanized and the aortic arch was excised for measuring radioactivity by storage phosphor imaging [18].

**Fig 6:**
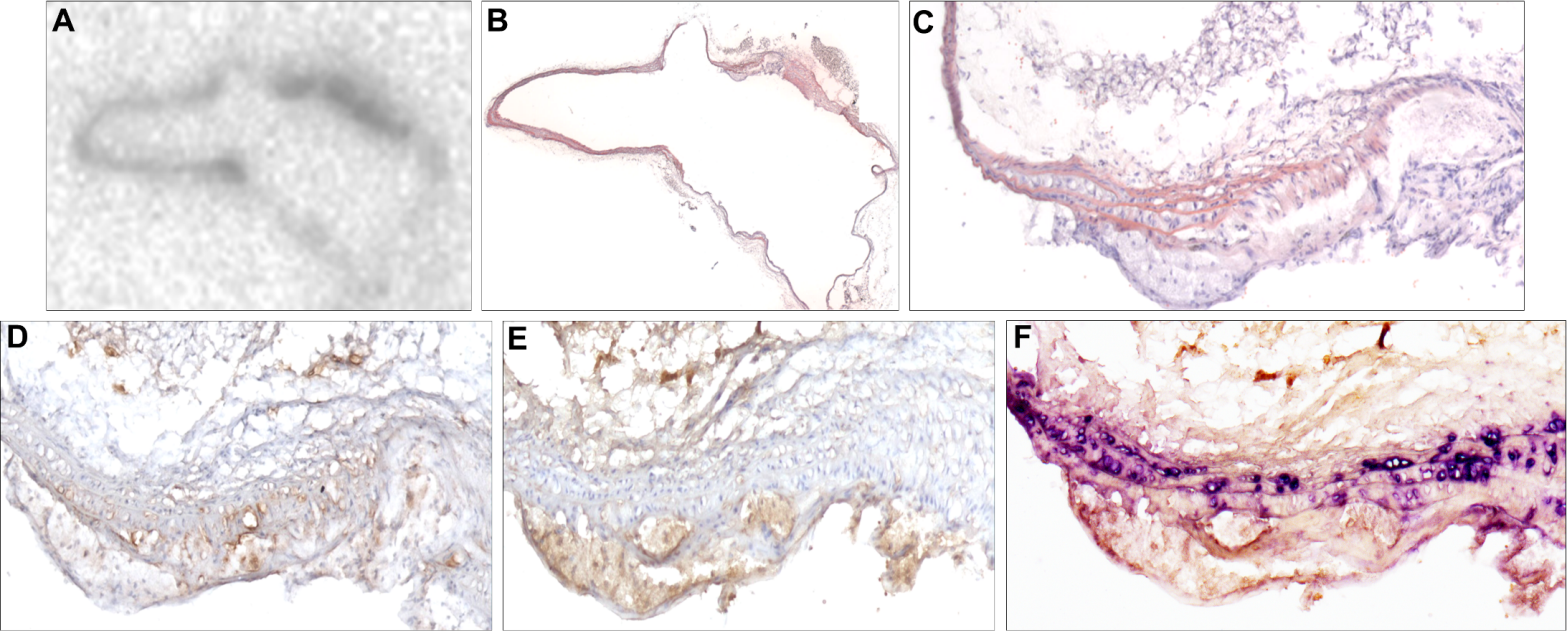
Binding of [^123^I]10c to atherosclerotic lesions containing MMP2 and MMP9. A) Storage phosphor image depicting binding of [^123^I]**10c** in a cross-section of the aortic arch of an ApoE-/- mouse. The region with highest apparent radioactivity has been outlined. B) Hematoxylin and eosin staining of the respective aortic arch cross-section shown in (A). The respective outlined region in (A) coincides with an atherosclerotic lesion at the base of a branching artery, as outlined in (B). C) Enlargement of the outlined region in (B). D) Cross-section of the atherosclerotic plaque in a consecutive section, displaying MMP2 (brown) distribution mainly in the smooth muscle cell-rich medial area of the aorta, and nuclei (blue). E) Cross-section of the atherosclerotic lesion in a consecutive section, depicting MMP9 (brown) distribution mainly in foam cell macrophages of the atherosclerotic plaque, and nuclei (blue). F) Respective section in (E) stained with anti-alpha-smooth muscle actin depicting smooth muscle rich layer (purple) in the aorta vessel wall, adjacent to the plaque core where apparent foam cells are present.

As depicted in Fig 6A, the uptake of [^123^I]**10c** was detectable in atherosclerotic lesions found in the aortic arch of ApoE-/- mice. The tracer uptake was relatively high in the diseased regions of the vessel wall. Fig 6B and 6C shows nuclear and cytoplasm staining of the respective histological section shown in Fig 6A, depicting the presence of anatherosclerotic lesion. To elucidate the selectivity of this radioligand towards MMP2 and MMP9, immunohistochemical staining revealed positive areas for MMP2 and MMP9 in the lesions co-localizing with uptake of [^123^I]**10c** that was detected by storage phosphor imaging (Fig 6D and 6E). This murine atherosclerotic lesion showed especially high MMP9 staining in the plaque (foam cell) macrophages, and not in the adjacent medial layer which consists of smooth muscle cells (Fig 6F). In conclusion, in this proof of concept study, the [^123^I]**10c** MMP2/9 tracer effectively targeted atherosclerotic lesions.

## Discussion

In this study we have developed a novel radiolabeled inhibitor of MMP2/9, which is able to target atherosclerotic lesions. The ligand we have derived in this study demonstrated an inhibitory potency in the desired nanomolar range, with a high selectivity in vitro for MMP2/9 over MMP1. We also demonstrated successful radiolabeling of the MMP inhibitor with [^123^I] using a single step approach. Thus, we have developed a novel highly potent molecular imaging agent for visualization of MMP2/9 activity by SPECT imaging, and thereby allowing for the identification of atherosclerotic lesions with high proteolytic MMP activity, which are considered vulnerable to rupture.

[^123^I]**10c** shows selectivity towards gelatinase MMP subtypes (MMP2 and MMP9) over MMP1. This is an important revelation, as previous broad spectrum inhibitors targeting MMP1 have led to adverse side effects due to interference with normal remodeling processes. We have demonstrated co-localization of [^123^I]**10c** in atherosclerotic lesions containing MMP2/9 in mice. This further highlights the feasibility to target MMP2/9-rich regions with the tracer and indicates that its accumulation in lesions is not due to vascular leakage.

The MMP inhibitor we have developed in this study displays highly potent inhibitory capacity in the range of 0.87 to 1.95 nM at MMP2/9. Although other existing MMP inhibitors display an even higher inhibitory potency [4, 14], these inhibitors did not show the high selectivity we have observed. Since MMP2/9 are typically expressed in areas under stress, such as atherosclerotic plaque lesions, the high selectivity for MMP2/9 will allow preferential targeting of atherosclerotic lesions.

We have selected iodine-123 as a suitable radionuclide to radiolabel compound **10c** as this would infer the compound appropriate for SPECT imaging. We selected a SPECT tracer due to the wider availability of SPECT machines, along with the convenient physical half-life of 13.2 hrs of the radionuclide. In addition, while PET scanners have superior sensitivity relative to SPECT imaging, SPECT cameras can detect different energies and thereby allow for simultaneous imaging of multiple radioisotopes [19, 20].

Radiolabeling of **10c** with iodine-123 was achieved successfully in a single step approach. This is advantageous to obtain the product with maximum radioactivity. In addition, stabilization of the compound in blood was noted approximately one hour after intravenous administration. Thus, the optimum time for imaging whereby signal-to-noise ratio is high is well within the limits of the half-life of iodine -123.

### Other applications of [^123^I]10c

As a result of the ECM remodeling capacity of MMPs and their participation in regulatory signaling in chronic inflammatory responses, these enzymes are involved in numerous pathologies, including cancer, arthritis in addition to atherosclerosis [21, 22]. The initial interest in MMP imaging, evolved from their role in cancer progression [12]. MMPs, particularly MMP2 and MMP9 modulate the degradation of the basal membrane, and thereby facilitate the migration of metastatic cells into proximal and distal tissues [23]. Thus, it is plausible that the MMP2 and MMP9 inhibitor developed in this study may have farther-reaching applications than only atherosclerotic lesion visualization and quantification. Nonetheless, future work must examine the efficacy of the ligand to also target metastatic tumors and allow visualization.

### Study limitations

Accumulation of [^123^I]**10c** in atherosclerotic lesions is not direct evidence of tracer binding to MMP2/9. However, taking into account that [^123^I]**10c** uptake co-localizes with regions of MMP2/9 immunostaining as well as the specific inhibitory potency towards MMP2/9, this accumulation of [^123^I]**10c** is suggestive of MMP2/9 binding. In addition, [^123^I]**10c** uptake has been visualized using phosphor imaging of excised tissue, rather than live *in vivo* SPECT imaging. Nevertheless, when combined with immunohistochemistry, this proof of concept method effectively demonstrates [^123^I]**10c** uptake in atherosclerotic lesions. Furthermore, although we have examined the inhibitory potency of our compound in relation to MMP2, 9 and 1, we cannot comment on the inhibitory potency of these compounds towards other MMP subtypes. We chose to examine the inhibitory potency of these compounds towards MMP1 in particular as previous broad spectrum MMP inhibitors have demonstrated severe side effects due to impairment of normal tissue remodeling that is regulated by MMP1. Nonetheless, as our compound **10a** is comparable to compound **B3** of Santos et al. we can speculate that it has similar inhibitory potencies for the other MMP subtypes. Santos et al. demonstrated that this compound shows highest inhibitory potency towards MMP2, MMP13 and MMP9 [14]. We are limited by a lack of metabolite assessment. *In vivo* biodistribution results of the tracer over time demonstrated a multi-phasic response in some tissues, suggesting the possibility of metabolite generation. Future studies should include examining the validation of this tracer in larger animal models, using animal SPECT imaging as well as examining the metabolic stability of the tracer. In addition, although we have examined the biodistribution of the tracer over time in blood, we did not examine its stability in plasma.

### Conclusions and future perspectives

We have developed a novel radioligand (*N*-[^123^I]iodophenylsulfonyl iminodiacetyl monohydroxamate) with high selectivity for MMP2/9 binding over MMP1. Nonetheless, as it is been published recently by Newby *et al*. [24] that other MMP subtypes also play a role in plaque rupture, the affinity to a larger panel of MMPs should be investigated in future studies. We have also demonstrated the efficacy of this tracer to target atherosclerotic lesions consisting of MMP2/9 in mice. Radiolabeling of **10c** was achieved using a single step approach, resulting in moderate radiochemical yield and high radiochemical purity. Biodistribution studies further revealed fast stabilization of the compound in blood. Nonetheless, our study is limited by a lack of metabolite detection. Future efforts will focus on further validation studies by *in vivo* SPECT imaging of mice prone to atherosclerotic lesion development along with the assessment for metabolite formation. Furthermore, it is imperative to examine the efficacy of these ligands in other species, with the ultimate realization of efficacy to target human MMP2/9. Development of tracers for visualization of atherosclerotic lesions prone to rupture, is an important step in the realization that atherosclerotic plaque complexity is an important indicator of the true risk of plaque rupture, rather than only angiographic assessment of atherosclerotic narrowing.

## Supporting information

S1 Appendix Supplemental methods describing chemical synthesis of analogues. (DOCX)

S2 Appendix ARRIVE guidelines checklist. (PDF)

S1 Fig **Synthesis of the arylsulfonylchlorides 7a-d from 5a-d** (i): ClSO_3_H, CH_2_Cl_2_, 0°C, 2h, (ii): SOCl_2_, DMF (cat), reflux, 6h, yield 80–97%.
(TIF)

S2 Fig **Attempted radiosynthesis of [**^**123**^**I]10c** (i): (*n*-Bu_3_Sn)_2_, Pd(PPh_3_)_4_, toluene, reflux, o/n, yield 72%; (ii): Na[^123^]I, H_2_O_2_, CH_3_COOH, rt, 20 min, yield 0%.
(TIF)

S3 Fig **Radiosynthesis of [**^**123**^**I]10c from 8c via electrophilic radioiodination.** (i): (*n*-Bu_3_Sn)_2_, Pd(PPh_3_)_4_, toluene, reflux, o/n, yield 76%; (ii): NaI[^123^], H_2_O_2_, CH_3_COOH, 25 min.; (iii): 2M HCl in Et_2_O, rt, 30 min.; (iv): a) ethylchloroformate, NMM, THF, 15 min, b) NH_2_OH x HCl, MeOH, 0°C, 15 min. Overall (steps ii to iv) decay corrected radiochemical yield of [^123^I]**10c** was 4%.
(TIF)

## Abbreviations

^3^*J*_HH_: Vicinal proton-proton coupling constant
δ: Chemical shifts
ACN: Acetonitrile
APCI: Atmospheric pressure chemical ionization
br s: Broad singlet
d: Doublet
DAB: 3,3’-diaminobenzidine
DCM: Dichloromethane
DMF: Dimethylformamide
DMSO-*d6*: Dimethyl sulfoxide-*d6*
ECF: Electrochemical fluorination
ESI: Electrospray ionization
EtOAc: Ethyl acetate
ECM: Extracellular matrix
HPLC: High performance liquid chromatography
IC_50_: Half maximal inhibitory concentration
IDA: Iminodiacetic acid
LC-MS: Liquid chromatography-mass spectrometry
m: Multiplet
MRI: Magnetic resonance imaging
MMPs: Matrix metalloproteinases
NMM: N-Methylmorpholine
NMR: Nuclear magnetic resonance
PET: Positron emission tomography
q: Quartet
R_f_: Retardation factor
s: Singlet
SD: Standard deviation
SEM: Standard error of the mean
SPECT: Single photon emission computed tomography
t: Triplet
TEA: Triethanolamine
THF: Tetrahydrofuran
TLC: Thin layer chromatography
UV: Ultraviolet
