# Supplementary material for: Novel molecular imaging ligands targeting matrix metalloproteinases 2 and 9 for imaging of unstable atherosclerotic plaques
Source: PLoS One. 2017 Nov 30;12(11):e0187767. doi: 10.1371/journal.pone.0187767 (PMC5708805; doi:10.1371/journal.pone.0187767)
Supplement: S1 Appendix — (DOCX) [file pone.0187767.s001.docx]

**Novel molecular imaging ligands targeting matrix metalloproteinases 2 and 9 for imaging of unstable atherosclerotic plaques**

Nazanin Hakimzadeh^1,2, ⁋^, Victorine A. Pinas^3,4, ⁋^, Ger Molenaar^5^, Vivian de Waard^6^, Esther Lutgens^6,7^, Berthe L.F. van Eck-Smit^3^, Kora de Bruin^3^, Jan J. Piek^2^, Jos L.H. Eersels^4^, Jan Booij^3^, Hein J. Verberne^3^, Albert D. Windhorst^4,*^

^1^Department of Biomedical Engineering & Physics, Academic Medical Center, University of Amsterdam, Amsterdam, The Netherlands

^2^Department of Cardiology, Academic Medical Center, Amsterdam, The Netherlands

^3^Department of Radiology and Nuclear Medicine, Academic Medical Center, University of Amsterdam, Amsterdam, The Netherlands

^4^Department of Radiology & Nuclear Medicine, VU University Medical Center, Amsterdam, The Netherlands

^5^BV Cyclotron VU, Amsterdam, The Netherlands

^6^Department of Medical Biochemistry, Academic Medical Center, Amsterdam, The Netherlands,

^7^Institute for Cardiovascular Prevention (IPEK) Ludwig Maximilian's University, Munich, Germany

^⁋^: N.H. and V.A.P. contributed equally to this article.

*Corresponding Author:

Email: [ad.windhorst@vumc.nl](mailto:ad.windhorst@vumc.nl) (ADW)

Short title: Radiolabeled MMP2/9 inhibitors for atherosclerotic plaque imaging

**Supplementary Methods**

**Supplementary Methods**

**Chemical Synthesis of analogues**

*Tert-butyl 2,2’-(phenylsulfonylazanediyl)diacetate (****2a****)*

To a solution of benzenesulfonyl chloride **1a** (1.68 g, 6.84 mmol) in dichloromethane (12 mL) was added triethylamine (0.63 g, 6.22 mmol), followed by a solution of *tert*-butyl 2,2’-azanediyl diacetate (1.10 g, 6.22 mmol) in dichloromethane (3 mL). After stirring at room temperature overnight, the solvent was evaporated. To the resulting residue H_2_O (15 mL) was added and the suspension was extracted with ethylacetate (4 × 20 mL). The combined organic extracts were washed with brine (2 × 20 mL), dried over anhydrous MgSO_4_, filtered and evaporated *in vacuo* to give the product as a light brown solid (1.82 g, 4.70 mmol, 75 %) *R_f_*_,_ 0.42 (hexane:ethylacetate 2:1). ^1^H-NMR (DMSO-*d6*) δ: 7.80-7.70 (m, 5H, *H*-2,3,4,5,6), 4.12 (s, 4H, C*H*_2_), 1.40 (s, 18H, C*H*_3_).^13^C-NMR (DMSO-*d6*) δ: 29.1 (CH_3_), 52.0 (CH_2_), 82.3 (C, C(CH_3_)_3_), 127.0 (CH, C-2/6), 129.0 (CH, C-3/5), 140.0 (CH, C-4), 141.3 (C, C-1), 169.1 (C, COO).

*Tert-butyl 2,2'-(4-fluorophenylsulfonylazanediyl)diacetate (****2b****)*

Compound **2b** was prepared employing a similar procedure as described for **2a** using 4-fluorobenzenesulphonyl chloride (**1b**) (1.00 g, 5.14 mmol). Yield: 0.76 g, 2.63 mmol, 56%. Light pink solid. *R_f_*_,_ 0.34 (hexane:ethylacetate 2:1). ^1^H-NMR (DMSO-*d6*) δ: 7.94 (m, 2H, *H*-2,6), 7.59 (m, 2H, *H*-3,5), 4.11 (s, 4H, C*H*_2_), 1.39 (s, 18H, C*H*_3_).

^13^C-NMR (DMSO-*d6*) δ: 28.7 (CH_3_), 51.4 (CH_2_), 81.0 (C, C (CH_3_)_3_), 116.0 (CH, C-2/6), 131.0 (CH, C-3/5), 135.3 (dd,^1^*J*­_CF_ = 243 Hz, ^2^*J*­_CF_ = 20 Hz, C, C-1), 167.5 (C, C-4), 171.0 (C, COO).

*Tert-butyl 2,2'-(4-iodophenylsulfonylazanediyl)diacetate (****2c****)*

Compound **2c** was prepared employing a similar procedure as described for **2a** using 4-iodobenzenesulphonyl chloride (1.00g, 3.31mmol). Yield: 0.520 g, 1.31 mmol, 40%, white solid. *R_f_*_,_ 0.39 (hexane:ethylacetate 2:1).

^1^H-NMR (DMSO-*d6*) δ: 8.12, (d, ^3^*J* = 8.2 Hz, 2H, *H*-2,6), 7.71 (d, ^3^*J* = 8.2 Hz, 2H, *H*-3,5), 4.10 (s, 4H, C*H*_2_), 1.38 (s,18H, C*H*_3_). ^13^C-NMR (DMSO-*d6*) δ: 29.0 (CH_3_), 50.1 (CH_2_), 82.0 (C(CH_3_)_3_), 98.2 (C, C-4), 128.9 (CH, C-2/6), 137.5 (CH, C-3/5), 138.2 (C, C-1), 171.3(C,COO).

*Tert-butyl 2,2'-(4-bromophenylsulfonylazanediyl)diacetate (****2d****)*

Compound **2d** was prepared employing a similar procedure as described for **2a** using 4-bromobenzenesulphonyl chloride (1.03 g, 4.03 mmol). Yield: 0.764 g, 1.65 mmol, 41%, light brown solid. *R_f_*_,_ 0.37 (hexane:ethylacetate 2:1). ^1^H-NMR (DMSO-*d6*) δ: 7.88-786, (m, 4H, *H*-2,3,5,6 ), 4.00 (s, 4H, C*H*_2_)*,* 1.35 (s, 18H, C*H*_3_). ^13^C-NMR (DMSO-*d6*) δ: 28.9 (CH_3_), 50.2 (CH_2_), 81.4 (C(CH_3_)_3_), 126.3 (CH, C-2/6), 129.5 (CH, C-3/5), 126.3 (C, C-4) 138.7 (C, C-1), 170.0 (C, COO).

*Tert-butyl 2,2'-(4-chlorophenylsulfonylazanediyl)diacetate (****2e****)*

Compound **2e** was prepared employing a similar procedure as described for **2a** using 4-chlorobenzenesulphonyl chloride (1.01 g, 4.74 mmol). Yield: 0.57 g, 1.85 mmol, 39% white solid. *R_f_*_,_ 0.46 (hexane:ethylacetate 2:1).^1^H-NMR (DMSO-*d6*) δ: 7.80, (d, ^3^*J* = 8.4 Hz, 2H, *H*-2,6), 7.64,(d, ^3^*J* = 8.4 Hz, 2H, *H*-3,5), 4.00 (s, 4H, C*H*_2_), 1.36 (s, 18H, C*H*_3_). ^13^C NMR (DMSO-*d6*) δ: 29.0 (CH_3_), 50.0 (CH_2_), 82.0 (C, C(CH_3_)_3_), 128.7 (CH, C-2/6), 130.5 (CH, C-3/5), 137.6 (C, C-1), 137.8 (C, C-4) 170.0 (C, COO).

*2,2’-(phenylsulfonylazanediyl)diacetic acid* *(****3a****)*

Compound **2a** (0.50 g, 5.85 mmol) was dissolved in formic acid (8 mL) and stirred overnight at room temperature. The precipitate was filtered and the filtrate was evaporated *in vacuo* and co-evaporated with toluene (6 × 10 mL). The product obtained was dried overnight in high vacuum. Yield: 0.487 g, 1.78 mmol, 30 %, white solid. *R_f_*_,_ 0.41, (hexane: ethylacetate 1:2). ^1^H-NMR (DMSO-*d6*) δ: 9.49(br s, 1H, O*H*), 7.80-7.70 (m, 5H, *H*-2,3,4,5,6), 4.12 (s, 4H, C*H*_2_*)*. ^13^C-NMR (DMSO-*d6*) δ: 51.0 (CH_2_), 130.2 (CH, C-3/5), 132.1 (CH, C-2/6), 132.1 (CH, C-4), 141.2 (C, C-1), 176.4 (C, COOH).

*2,2’-(4-fluorophenylsulfonylazanediyl)diacetic acid* *(****3b****)*

Compound **3b** was prepared employing a similar procedure as described for **3a** using compound **2b** (0.760 g, 2.63 mmol). Yield: 0.700 g, 2.40 mmol, 91%, light yellow solid. *R_f_*_,_ 0.41 (hexane:ethylacetate 1: 2).

^1^H-NMR (DMSO-*d6*) δ: 13.01 (br s, 2H, O*H*), 7.94 (m, 2H, *H*-2/6, HF), 7.59 (m, *H*-3/5), 4.11 (s, 4H, C*H*_2_). ^13^C-NMR (DMSO-*d6*, δ): 28.7 (CH_3_), 51.0 (CH_2_), 116.0 (CH, C-3/5), 130.4 (CH, C-2/6), 135.3 (135.3 (dd,^1^*J*­_CF_ = 240 Hz, ^2^*J*­_CF_ = 22 Hz, C, C-1),, 167.2 (C, C-4), 174.0 (C, COOH).

*2,2’-(4-iodophenylsufonylazanediyl) diacetic acid* *(****3c****)*

Compound **3c** was prepared employing a similar procedure as described for **3a** using compound **2c** (0.52 g, 1.31 mmol). Yield: 0.479 g, 1.20 mmol, 92 %, white solid. *R_f_*_,_ 0.37 (hexane:ethylacetate 1: 2).

^1^H-NMR (DMSO-*d6*) δ: 8.12 (d, ^3^*J* = 8.4 Hz, 2H, *H*-2/6), 7.71 (d, ^3^*J* = 8.4 Hz, 2H, *H*-3/5), 4.00 (s, 4H, C*H*_2_). ^13^C-NMR (DMSO-*d6* δ) δ: 52.0 (CH_2_), 97.4 (C, C-4), 129.1 (CH, C-2/6), 137.2 (CH, C-3/5), 138.4 (C, C-1), 173.4 (C, COOH).

*2,2’-(4-bromophenylsulfonylazanediyl) diacetic acid* *(****3d****)*

Compound **3d** was prepared employing a similar procedure as described for **3a** using compound **2d** (0.76g, 1.65 mmol). Yield: 0.54 g, 1.54 mmol, 93%, light brown solid. *R_f_*_,_ 0.41 (hexane:ethylacetate 1: 2).

^1^H-NMR (DMSO-*d6*) δ: 7.88-786 (m, 4H, *H*-2,3,5,6), 4.00 (s, 4H, C*H*_2_). ^13^C-NMR (DMSO-*d6*) δ: 51.4 (CH_2_), 130.0 (CH, C-2/6), 132.1 (CH, C-3/5), 126.7 (C, C-4) 138.7 (C, C-1), 176.2 (C, COOH).

*2,2’-(4-chlorophenylsulfonylazanediyl)diacetic acid* *(****3e****)*

Compound **3e** was prepared employing a similar procedure as described for **3a** using compound **2e** (0.570g, 1.85 mmol). Yield: 0.473 g, 1.54 mmol, 83 %, white solid. *R_f_*_,_ 0.39 (hexane:ethylacetate 1:2).

^1^H-NMR (DMSO-*d6*) δ: 7.80 (d, ^3^*J* = 8.4 Hz, 2H, *H*-2,6), 7.64 (d, ^3^*J* = 8.4 Hz, 2H, *H*-3,5), 4.11 (s, 4H, C*H*_2_). ^13^C-NMR (DMSO-*d6*) δ: 52.2 (CH_2_), 128.3 (CH, C-2/6), 129.4 (CH, C-3/5), 137.3 (C, C-1), 138.0 (C, C-4) 170.0 (C, COOH).

*2-(N-(2(hydroxyamino)-2-oxoethyl)phenylsulfonamido) acetic acid (****4a****)*

Compound **3a** (0.487g, 1.78 mmol) was dissolved in dry THF (10 mL). Ethylchloroformate (0.193g, 1.78 mmol), and *N*-methylmorpholine (0.180g 1.78 mmol) were added and this mixture was stirred for 40 minutes at 0 °C. The hydroxylamine hydrochloric acid salt (0.12 g, 1.78 mmol) and KOH (0.10 g, 1.78 mmol) were dissolved in dry MeOH (5 mL) and stirred at 0 °C for 30 minutes. The precipitate was filtered off and the THF solution was dropwise added to the methanol filtrate and stirred for 2 hours at 0 °C. The reaction mixture was filtered, the solvent was evaporated and the residue was dissolved in H_2_O (10 mL). After adjusting the pH using a 1% hydrochloric acid solution to pH 1-2, the aqueous layer was extracted with EtOAc (3 × 10 mL). The combined organic layers were dried over MgSO_4_, evaporated *in vacuo* and co‑evaporated with toluene (3 × 3 mL) to yield the product. Yield: 0.498 g, 1.73 mmol, 97 %, white solid. *R_f_*_,_ 0.32 (hexane:ethylacetate 4:1).

^1^H-NMR (DMSO-*d6*) δ: 7.87-7.71 (m, 5H, *H*-2,3,4,5,6), 4.11 (s, 2H, CH_2_), 4.04 (s, 2H, C*H*_2_). ^13^C-NMR (DMSO-*d6*) δ: 42.8 (CH_2_), 51.3 (CH_2_), 127.3 (CH, C-3/5), 129.0 (CH, C-2/6), 132.0 (CH, C-4), 140.1 (C, C-1), 167.5 (C, CONHOH), 172.5 (C, COOH). LC-MS (ESI^+^): Found 289.367 (M+H) (calc. for C_10_H_11_NO_6_S 288.0); 229.1, 169.1, 60.0. Elemental analysis (C, H, N) calculated (C42.1%, H 4.50%, N 9.97%) found (C 42.0%, H 4.20%, N 9.72%).

*2-(4-fluoro-N-(2-hydroxyamino)-2-oxoethyl)phenylsufonamido acetic acid (****4b****)*

Compound **4b** was prepared following the procedure described for **4a** with reagent quantities adapted to the amount of starting material **3b** (0.700 g, 2.40 mmol). Yield 0.657g, 2.15 mmol, 89%, light yellow solid. *R_f_*_,_ 0.39 (hexane:ethylacetate 4: 1).

^1^H-NMR (DMSO-*d6*) δ: 7.94 (m 2H, *H*-3,5), 7.59 (2H, *H*-2,6), 4.11 (s, 2H, C*H*_2_), 4.01 (s, 2H, CH_2_). ^13^C-NMR (DMSO-*d6*) δ: 42.1 (CH_2_), 51.0 (CH_2_), 115.2 (CH, C-3,5), 131.0 (CH, C-2,6), 135.4 (dd,^1^*J*­_CF_ = 241 Hz, ^2^*J*­_CF_ = 20 Hz ,C, C-1), 167.1 (C, C-4), 168.6 (C, CONHOH ), 174.0 (C, COOH). LC-MS (ESI^+^): Found 307.269 (M+H) (calc. for C_10_H_10_FN_2_O_6_S306.261), 247.2, 187.2, 60.0. Elemental analysis (C,H,N) calculated (C 40.0%, H 3.65%, N 9.10%) found (C 39.9%, H 3.64%, N 9.12%).

*2-(4-Iodo-N-(2-hydroxyamino)-2-oxoethyl) phenylsufonamido acetic acid (****4c****)*

Compound **4c** was prepared following the procedure described for **4a** from **3c** (0.479 g, 1.20 mmol). Yield 0.401g, 0.96 mmol, 80%, light brown solid. *R_f_*_,_ 0.31 (hexane:ethylacetate 4:1).

^1^H-NMR (DMSO-*d6*) δ: 8.10 (d, ^3^*J* = 8.2 Hz, 2H, *H*-2,6), 7.71 (d, ^3^*J* = 8.2 Hz, 2H, *H*-3,5), 4.06 (s, 4H, C*H*_2_). 4.00 (s, 4H, C*H*_2_). ^13^C-NMR (DMSO-*d6*) δ: 41.2 (CH_2_), 50.9 (CH_2_), 95.7 (C, C-4), 129.0 (CH, C-2/6), 138.5 (CH, C-3/5), 139.7 (C, C-1), 167.8 (C, CONHOH),176.9 (C, COOH). LC-MS (ESI^+^) Found 415.101 (M+H) (calc. for C_10_H_11_IN_2_O_6_S 414.9), 354.2, 228.2, 168.2, 59.9**.** Elemental analysis (C,H,N) calculated (C 29.0%, H 2.60%, N 6.76%) found (C 29.1%, H 2.71%, N 6.45 %).

*2-(4-bromo-N-(2-hydroxyamino)-2-oxoethyl) phenylsufonamido acetic acid (****4d****)*

Compound **4d** was prepared following the procedure described for **4a** from **3d** (0.543 g, 1.54 mmol). Yield: 0.358 g, 0.98 mmol, 63%, white solid. *R_f_*_,_ 0.33 (hexane:ethylacetate 4:1).

^1^H-NMR (DMSO-*d6*) δ: 7.90-787 (m, 4H, *H*-2,3,5,6 ), 4.11 (s, 2H, C*H*_2_), 4.00 (s, 2H, C*H*_2_). ^13^C-NMR (DMSO-*d6* δ) δ: 42.0 (CH_2_), 52.1 (CH_2_), 129.3 (CH, C-4), 130.0 (CH, C-3/5), 132.4 (C, C-2/6), 139.0 (C, C-1), 167.2 (C, CONHOH).), 175.8 (C, COOH). LC-MS (ESI^+^) Found 367.952 and 365.954 (M+H) (calc. for C_10_H_11_BrN_2_O_6_S 367.9 and 365.9), 308.4, 248.3, 60.0 and 366.6, 307.0, 247.0. Elemental analysis (C,H,N) calculated (C 32.71%, H 3.02%, N 7.63%) found (C 32.78%, H 3.01%, N 7.97%).

*2-(4-chloro-N-(2-hydroxyamino)-2-oxoethyl)phenylsufonamido acetic acid (****4e****)*

Compound **4e** was prepared following the procedure described for **4a** from **3e** (0.47 g, 1.54 mmol). Yield: 0.345 g, 1.07 mmol, 69% white solid. *R_f_*_,_ 0.34 (hexane:ethylacetate 4: 1).

^1^H-NMR (DMSO-*d6*) δ: 7.80 (d, ^3^*J* = 8.4 Hz, 2H, *H*-2,6), 7.64 (d, ^3^*J* = 8.4 Hz, 2H, *H*-3,5), 4.10 (s, 2H, C*H*_2_), 4.03 (s, 2H, C*H*_2_). ^13^C-NMR (DMSO-*d6*) δ: 41.1 (CH_2_), 50.9 (CH_2_), 128.7 (CH, C-2/6), 129.2 (CH, C-3/5), 137.4 (C, C-1), 137.2 (C, C-4), 174.6 (C, CONHOH).), 176.4 (C, COOH). LC-MS (ESI^+^): Found 323.763 and 325.810 (M+H) (calc. for C_10_H_11_ClN_2_O_6_S 322.0), 263.7, 203.7, 60.0**.**  Elemental analysis (C, H, N) calculated (C 37.22%, H 3.22%, N 8.68%) found (C 37.23%, H 3.46%, N 8.69%).

*4-Phenoxy‑benzenesulphonyl chloride* *(****7a****)*

To a solution of diphenylether **5a** (2.00 g, 11.8 mmol) in dry dichloromethane (30 mL) under an argon atmosphere at 0 °C, was added chlorosulphonic acid (0.80 mL, 11.8 mmol). The reaction mixture was stirred for 2 hours at 0 °C. The solvent was evaporated at room temperature and the residue was dried overnight under vacuum to give the corresponding 4-phenoxybenzenesulphonic acid **6a** as pink oil. Compound **6a** was used for the next reaction without further purification. The sulphonic acid **6a** was dissolved in an excess of thionylchloride (30 mL), a catalytic amount of DMF (0.2 mL) was added, and the resulting mixture was stirred for 6 hours at reflux. The solvent was evaporated and the residue dissolved in diethylether (40 mL). The solution was washed with 5 % aqueous NaOH (3 × 40 mL) and H_2_O (6 × 40 mL), dried over anhydrous MgSO_4_, filtered, and concentrated *in vacuo.* After drying in high vacuum overnight, the pure product was obtained as a white solid. Yield: 2.09 g, 7.44 mmol, 63%, brown oil. *R_f_* 0.37 (hexane:ethylacetate 3:1).

^1^H-NMR (DMSO-*d6*) δ: 7.92 (d, ^3^*J* = 8.9 Hz, 2H, *H*-2/6), 7.87 (m*,* 1H, *H*-4’), 7.38 (d, ^3^*J*_HH_ = 8.9 Hz, 2H, *H*-3/5), 7.12 (d, ^3^*J* = 9.1 Hz, 2H, *H*-2’ /6’), 6.98 (m, 2H, *H*-3’/5’). ^13^C-NMR (DMSO-*d6*) δ: 121.8 (CH, C-2/6), 128.4 (CH, C-3/5), 128.5 (CH, C-2’/6’), 128.7 (CH, C-3’, 4’, 5’), 137.4 (C, C-1), 157.0 (C, C-1’), 163.7 (C, C-4).

*4‑ (4‑Fluoro‑phenoxy)‑benzenesulphonyl chloride (****7b****)*

Compound **7b** was prepared similar to the procedure described for **7a** using (4‑fluorodiphenyl) ether (1.00 g, 5.31 mmol). Yield 1.04 g, 3.63 mmol, 75%, white solid. *R_f_* 0.32 (hexane:ethylacetate 3:1).

^1^H-NMR (DMSO-*d6*) δ: 7.99 (d, ^3^*J*­ = 9,1 Hz, 2H, *H*-2,6), 7.59 (d, ^3^*J_HH_*­ = 9.1 Hz, 2H, *H*-3,5), 7.38 (m, 2H, *H*-2’,6’), 6.94 (m, 2H, *H*-3’,5’). ^13^C-NMR (DMSO-*d6*) δ: 121.8 (CH, C-2,6), 128.4 (CH, C-3,5), 128.5 (CH, C-2’,6’), 128.7 (CH, C-3’,5’), 137.4 (dd,^1^*J*­_CF_ = 238 Hz, ^2^*J*­_CF_ = 24 Hz, C, C-1), 156.0 (C, C-4’), 157.0 (,C, C-1’), 163.8 (C, C-4).

*4-(4-Iodo-phenoxy)-benzenesulfonyl chloride (****7c****)*

Compound **7c** was prepared similar to the procedure described for **7a** using (4-iododiphenyl) ether (1.12 g, 3.78 mmol). Yield 1.20 g, 3.03 mmol, 80 %, light pink solid. *R_f_* 0.32 (hexane:ethylacetate 3:1).

^1^H-NMR (DMSO-*d6*) δ: 7.85, (d, ^3^*JHH* = 8.9 Hz, 2H, *H*-2,6), 7.76 (d, ^3^*J*_HH_ = 8.9 Hz, 2H, *H*-3,5), 7.63 (d, ^3^*J*_HH_ = 8.9 Hz, 2H, *H*-3’,5’), 6.86 (d, ^3^*J*_HH_ = 8.9 Hz, 2H, *H*-2’,6’). ^13^C-NMR (DMSO-*d6* δ) δ: 89.6 (C, C-4’), 118.9 (CH, C-3,5), 119.3 (CH, C-2’,6’), 128.7 (CH, C-2,6), 135.7 (CH, C-3’,5’), 137.4 (C, C-1), 154.7 (C, C-1’), 164.1 (C, C-4).

*4-(4-Bromo-phenoxy)-benzenesulphonyl chloride (****7d****)*

Compound **7d** was prepared similar to the procedure described for **7a** using 4‑bromodiphenyl ether (2.00 g, 8.04 mmol). Yield: 2.70 g, 7.80 mmol, 97 %, white solid. *R_f_* 0.41 (hexane:ethylacetate 3:1).

^1^H-NMR (DMSO-*d6*) δ: 7.95 (d, ^3^*J*_HH_ = 8.9 Hz, 2H, *H*-2,6), 7.65 (d, ^3^*J*_HH_ = 8.8Hz, 2H, *H*-3,5), 7.51-7.40 (m, 4H, *H*-2’,3’,5’,6’). ^13^C-NMR (DMSO-*d6*) δ: 115.9 (CH, C-2’,6’),116.4 (C, C-4’), 118.9 (CH, C-3,5), 128.6 (CH, C-2,6), 131.5 (CH, C-3’,5’), 137.4 (C, C-1), 156.1 (C, C-1’), 164.1 (C, C-4).

*Tert-butyl 2, 2’-(4-phenoxyphenylsulfonylazanediyl) diacetate (****8a****)*

To a solution of di-O-*t*Bu-protected IDA (1.64g, 6.67 mmol) in dichloromethane (15 mL) was added triethylamine (0.67g, 6.67 mmol), followed by a solution 4-phenoxy‑benzenesulphonyl chloride (2.00 g, 7.44 mmol) in dichloromethane (3 mL). After stirring at room temperature overnight, the white solid was filtered off, and the filtrate was evaporated. To the resulting residue was added H_2_O (15 mL) and the suspension was extracted with EtOAc (4 × 40 mL). The combined organic extracts were washed with brine (2 × 20 mL), dried over anhydrous MgSO_4_, filtered and evaporated *in vacuo* to give the pure product. Yield: 2.78 g, 78%, brown oil. *R_f_* 0.41 (hexane:ethylacetate 3:1).

^1^H-NMR (DMSO-*d6*) δ: 7.71 (d, ^3^*J*_HH_ = 9.0 Hz, 2H, *H*-3’,5’),7.41 (d, 2H, 2/6), 7.38 (d, ^3^*J*_HH_ = 9.0 Hz, 2H, *H*-2’,6’), 7.18 ( m, 1H, C-4), 7.14 (d, ^3^*J*_HH_ = 9.1 Hz, 2H, *H*-3,5’, 4.10 (s, 4H, C*H*_2_) 1.41 (s, 18 H, C*H*_3_). ^13^C-NMR (DMSO-*d6*) δ: 29.1 (CH_3_), 49.3 (CH_2_), 81.2 (C, C(CH­_3_)_3_), 117.0 (CH, C-3,5), 119.3 (CH, C-2’,6’), 122.3 (CH, C-4’), 124.7 (CH, C-2,6),128.6 (CH, C-3’,5’), 133.6 (C, C-1), 158.3 (C, C-1’), 161.2 (C, C-4), 169.5 (C, COO).

*[4-(4-Fluorophenoxy)-benzenesulphonyl]-tert-butoxycarbonylmethyl-amino-aceticacid tert-butyl ester (****8b****)*

Compound **8b** was prepared employing a similar procedure as described for **8a** using 4-(4-fluorophenoxy)-benzenesulphonyl chloride (0.64 g, 2.34 mmol). Yield: 1.01 g, 2.03 mmol, 99 %, white solid. *R_f_* 0.37 (hexane:ethylacetate 3:1).

^1^H-NMR (DMSO-*d6*) δ: 7.91 (m, 2H, *H*-2,6, HF), 7.43 (m,2H, *H*-3,5), 7.35 (d, ^3^*J*_HH_ = 8.2 Hz, 2H, *H*-2’,6’), 7.21 (d, ^3^*J*_HH_ = 8.2 Hz, 2H, *H*-3’,5’), 4.05 (s, 4H, C*H*_2_), 1.41 (s, 18 H, C*H*_3_). ^13^C-NMR (DMSO-*d6*) δ: 28.7 (CH_3_), 50.3 (CH_2_), 82.0 (C(CH_3_)_3_), 115.2 (CH, C-2’,6’), 117.8 (CH, C-3,5), 120.3 (CH, C-3’,5’), 124.8 (CH, C-2,6), 132.8 (dd,^1^*J*­_CF_ = 242 Hz, ^2^*J*­_CF_ = 21 Hz, C, C-1), 152.4 (C, C-4’), 158.0 (C, C-4), 161.7 (C, C-1’), 171.4 (C, COO).

*([4-(4-Iodophenoxy)-benzenesulphonyl]-tert-butoxycarbonylmethyl-amino) acetic acid tert-butyl ester (****8c****)*

Compound **8c** was prepared following the procedure described for **8a** using 4-(4-iodophenoxy) benzenesulphonyl chloride (0.71 g, 1.81 mmol). Yield: 1.04 g, 1.64 mmol, 99 %, white solid. *R_f_*_,_ 0.37 (hexane:ethylacetate 3:1).

^1^H-NMR (DMSO-*d6* δ) δ: 7.82, (d, ^3^*J*_HH_ = 8.7 Hz, 2H, *H*-2,6), 7.69 (d, ^3^*J*_HH_ = 8.7 Hz, 2H, *H*-3,5), 7.02 (d, ^3^*J*_HH_ = 8.9 Hz, 2H, *H*-3’,5’), 6.82 (d, ^3^*J*_HH_ = 8.9 Hz, 2H, *H*-2’,6’), 4.09 (s, 4H, C*H*_2_), 1.43 (s, 18 H, C*H*_3_).

^13^C-NMR (DMSO-*d6*) δ: 28.9 (CH_3_), 50.7 (CH_2_), 81.1 (C(CH_3_)_3_), 85.3 (C, C-4’), 117.4 (CH, C-3,5), 119.4 (CH, C-2’,6’), 124.9 (CH, C-2,6), 132.3 (C, C-1), 137.3 (CH, C-3’,5’), 155.9 (C, C-1’), 160.2 (C, C-4), 171.4 (C, COO).

*([4-(4-Bromophenoxy)-benzenesulphonyl]-tert-butoxycarbonylmethyl-amino) acetic acid tert-butyl ester (****8d****)*

Compound **8d** was prepared similar to the procedure described for **8a** using 4-(4-bromophenoxy)-benzenesulphonyl chloride (3.00 g, 8.63 mmol). Yield: 4.13 g, 86 %, light yellow solid. *R_f_*_,_ 0.39 (hexane: ethylacetate 3:1).

^1^H-NMR (DMSO-*d6*) δ: 7.95 (d, ^3^*J*_HH_ = 8.4 Hz, 2H, *H*-2,6), 7.65 (d, ^3^*J*_HH_ = 8.4 Hz, 2H, *H*-3,5), 7.50-7.42 (m, 4H, *H*-2’,3’,5’,6’), 4.05 (s, 4H, C*H*_2_), 1.46 (s, 18 H, C*H*_3_). ^13^C-NMR (DMSO-*d6*) δ: 28.7 (CH_3_), 50.4 (CH_2_), 81.9 (C(CH_3_)_3_), 117.4 (CH, C-2’,6’), 116.0 (C, C-4’), 118.1 (CH, C-3,5), 124.8 (CH, C-2,6), 131.3 (CH, C-3’,5’), 132.8 (C, C-1), 156.0 (C, C-1’), 160.4 (C, C-4), 156.0 (C, COO).

*([4-Phenoxy-benzenesulphonyl]-carboxymethyl-amino) acetic acid* *(****9a****)*

Compound **8a** (1.02 g, 2.14 mmol) was dissolved in formic acid (10 mL) and stirred overnight at room temperature. The solid was filtered and the filtrate was evaporated *in vacuo* and co-evaporated with toluene (6 × 20 mL). The pure product was dried overnight in high vacuum. Yield: 0.71 g, 1.95 mmol, 91 %, off-white solid. *R_f_* 0.37 (hexane:ethylacetate 1:3). ^1^H-NMR (DMSO-*d6*) δ: 7.91 (d, ^3^*J*_HH_ = 9.3 Hz, 2H, *H*-2,6), 7.41 (m*,* 1H, *H*-4’), 7.20 (d, ^3^*J*_HH_ = 8.6 Hz, 2H, *H*-2’,6’), 7.14 (d, ^3^*J*_HH_ = 9.3 Hz, 2H, *H*-3,5), 6.87 (t, ^3^*J*_HH_ = 8.6 Hz, 2H, *H*-3’,5’), 4.07 (s, 4H, C*H*_2_). ^13^C-NMR (DMSO-*d6*, δ): 51.9 (CH_2_), 117.9 (CH, C-3,5), 118.4 (CH, C-2’,6’), 124.8 (CH, C-2,6), 128.4 (CH, C-3’,5’), 132.7 (C, C-1), 158.3 (C, C-1’), 121.6 (CH, C-4’), 161.4 (C, C-4), 180.1 (C, COOH).

*([4-(4-Fluorophenoxy)-benzenesulphonyl]-carboxymethyl-amino) acetic acid* *(****9b****)*

Compound **9b** was prepared from **8b** (1.00 g, 2.03 mmol) using the procedure described for **9a.** Yield: 0.68 g, 1.78 mmol, 88 %, off-white solid. *R_f_* 0.32 (hexane:ethylacetate 1:3).

^1^H-NMR (DMSO-*d6*) δ: 7.72 (d, ^3^*J*_HH_ = 8.7 Hz, 2H, *H*-2,6), 7.47 (d, ^3^*J*_HH_ = 8.7 Hz, 2H, *H*-3,5), 7.37 (m, 2H, *H*-2’,6’), 7.22 (m, 2H, *H*-3’,5’), 4.09 (s, 4H, C*H*_2_). ^13^C-NMR (DMSO-*d6*) δ: 52.0 (CH_2_), 115.2 (CH, C-2’,6’), 120.4 (CH, C-3,5), 117.9 (CH, C-3’,5’), 124.1 (CH, C-2,6), 132.8 (C, C-1), 156.0 (C, C-4’), 152.6 (C, C-4), 160.8 (dd,^1^*J*­_CF_ = 241 Hz, ^2^*J*­_CF_ = 22 Hz,C, C-1’), 179.4 (C, COOH).

*([4-(4-Iodo-phenoxy)-benzenesulphonyl]-carboxymethyl-amino) acetic acid* *(****9c****)*

Compound **9c** was prepared following the procedure described for **9a** with reagent quantities adapted to the amount of starting material **8c** (0.68 g, 1.08 mmol). Yield: 0.50 g, 1.02 mmol, 94%, white solid. *R_f_* 0.35 (hexane:ethylacetate 1:3). ^1^H-NMR (DMSO-*d6*) δ: 7.82, (d, ^3^*J*_HH_ = 8.8 Hz, 2H, *H*-2,6), 7.64 (d, ^3^*J*_HH_ = 8.8 Hz, 2H, *H*-3,5), 7.17 (d, ^3^*J*_HH_ = 8.4 Hz, 2H, *H*-3’,5’), 7.06 (d, ^3^*J*_HH_ = 8.4 Hz, 2H, *H*-2’,6’), 4.09 (s, 4H, C*H*_2_). ^13^C-NMR (DMSO-*d6*) δ: 51.3 (CH_2_), 84.2 (C, C-4’), 117.4 (CH, C-3,5), 119.6 (CH, C-2’,6’), 124.6 (CH, C-2,6), 132.8 (C, C-1), 137.3 (CH, C-3’,5’), 155.7 (C, C-1’), 161.0 (C, C-4), 174.8 (C, COO).

*([4-(4-Bromophenoxy)-benzenesulphonyl]-carboxymethyl-amino) acetic acid (****9d****)*

Compound **9d** was obtained from **8d** (1.00g, 1.80 mmol) following the procedure described for **9a**. Yield: 0.60g, 1.35mmol, 75%, white solid. *R_f_* 0.37 (hexane:ethylacetate 1:3). ^1^H-NMR (DMSO-*d6*) δ: 7.82 (d, ^3^*J*_HH_ = 8.8 Hz, 2H, *H*-2,6), 7.64 (d, ^3^*J*_HH_ = 8.8 Hz, 2H, *H*-3,5), 7.48 -7.34 (m, 4H, *H*-2’,3’,5’,6’), 3.96 (s, 4H, C*H*_2_). ^13^C-NMR (DMSO-*d6*) δ: 51.1 (CH_2_), 117.4 (CH, C-2’,6’), 116.0 (C, C-4’), 118.2 (CH, C-3,5), 124.8 (CH, C-2,6), 131.4 (CH, C-3’,5’), 131.4 (C, C-1), 156.0 (C, C-1’), 160.4 (C, C-4), 180.1 (C, COOH).

*([4-Phenoxy-benzenesulphonyl]-hydroxycarbamoylmethyl-amino) acetic acid* *(****10a****)*

Compound **9a** (0.71 g, 1.95 mmol) was dissolved in dry THF (5 mL). ECF (160 μL, 1.95 mmol) and NMM (180 μL, 1.95 mmol) were added and stirred for 40 minutes at 0 °C. NH_2_OH•HCl (136 mg, 1.95 mmol) and KOH (109 mg, 1.95 mmol) were dissolved in dry MeOH (5 mL) and stirred at 0 °C for 30 minutes. The solids were filtered off and the THF solution was added dropwise to the methanol filtrate and stirred for 2 hours at 0 °C. The reaction mixture was filtered, the solvent evaporated and the residue dissolved in H_2_O (25 mL). After adjusting the pH to 1-2, the aqueous layer was extracted with EtOAc (3 × 30 mL). The combined organic layers were dried over MgSO_4_, evaporated *in vacuo* and co-evaporated with EtOAc (3 × 3 mL). Yield: 0.565 g, 1.10 mmol, 56%, light-pink solid. *R_f_* 0.42 (hexane:ethylacetate 4:1).

^1^H-NMR (DMSO-*d6*) δ: 7.87 (d, ^3^*J*_HH_ = 8.6 Hz, 2H, *H*-2,6), 7.42 (m*,* 1H, *H*-4’), 7.35 (d, ^3^*J*_HH_ = 8.6 Hz, 2H, *H*-3,5), 6.98 (d, ^3^*J*_HH_ = 9.3 Hz, 2H, *H*-2’,6’), 6.90 (m, 2H, *H*-3’,5’), 4.07 (s, 2H, C*H*_2_), 3.85 (s, 2H, C*H*_2_). ^13^C-NMR (DMSO-*d6*) δ: 42.6 (CH_2_COOH), 51.3 (CH_2_NHOH), 117.5 (CH, C-3,5), 118.7 (CH, C-2’,6’), 121.6 (CH, C-4’), 124.3 (CH, C-2,6), 128.4 (CH, C-3’,5’), 132.7 (C, C-1) 158.3 (C, C-1’), 160.1 (C, C-4), 165.4 (C, CONHOH), 175.8 (C, COOH). LC-MS (ESI^+^): Found 381.472 (M+H), (calc. for C_16_H_16_N_2_O_7_S MH, 380.1); 304.5, 244.4, 184.4, 77.0. Element analysis (C, H, N) Calculated (C 50.5%, H 4.23%, N 7.36%) found (C 50.4%, H 4.22%, N 7.96 %).

*([4-(4-Fluoro-phenoxy)-benzenesulphonyl]-hydroxycarbamoylmethyl-amino) acetic acid* *(****10b****)*

Compound **10b** was prepared following the procedure described for **10a** with reagent quantities adapted to the amount of starting material **9b** (500 mg, 1.30 mmol). Yield: 267 mg, 0.67 mmol, 52 %, white solid. *R_f_* 0.37 (hexane:ethylacetate 4:1).

^1^H-NMR (DMSO-*d6*) δ: 7.91(d, ^3^*J*_HH_ = 9.0 Hz, 2H, *H*-2,6), 7.42 (d, ^3^*J*_HH_ = 9.0 Hz, 2H, *H*-3,5), 6.99 (m, *H*-2’,6’), 6.09 (m, ^3^*J*_HH_ = 9.1 Hz 2H, *H*-3’,5’), 4.03 (s, 4H, C*H*_2_). ^13^C-NMR (DMSO-*d6*) δ: 42.4 (CH_2_COOH), 51.7 (CH_2_NHOH), 116.9 (CH, C-2’,6’), 117.4 (CH, C-3’,5’), 120.8 (CH, C-3,5), 124.7 (CH, C-2,6), 153.8 (dd,^1^*J*­_CF_ = 241 Hz, ^2^*J*­_CF_ = 20 Hz,C, C-1), 159.7 (C, C-4’), 156.4 (C, C-4), 160.2 (C, C-1’), 166.5 (C, CONHOH), 176.8 (C, COOH).

LC-MS (ESI^+^ ): Found 398.061 (M+H), (calc. for C_16_H_15_FN_2_O_7_S 398.1); 322.1,303.1, 243.1, 183.1, 77.2. Elemental analysis (C,H,N) calculated (C 48.2, H 3.80%, N 7.03%) found (C 48.3%, H. 3.79%, N.7.02 %).

*([4-(4-Iodo-phenoxy)-benzenesulphonyl]-hydroxycarbamoylmethyl-amino) acetic acid (****10c****)*

Compound **10c** was prepared following the procedure described for **10a** with reagent quantities adapted to the amount of starting material **9c** (500 mg, 1.02 mmol). Yield: 305 mg, 0.60 mmol, 59 %, White solid. *R_f_* 0.35 (hexane:ethylacetate 4:1).

^1^H-NMR (DMSO-*d6*) δ: 7.72 (d, ^3^*J*_HH_ = 8.2 Hz, 2H, *H*-2,6), 7.52 (d, ^3^*J*_HH_ = 8.2 Hz, 2H, *H*-3,5), 7.03 (d, ^3^*J* = 8.8 Hz, 2H, *H*-3’,5’), 6.65 (d, ^3^*J*_HH_ = 8.8 Hz, 2H, *H*-2’,6’), 4.01 (s, 4H, C*H*_2_), 3.93 (s, 4H, C*H*_2_). ^13^C-NMR (DMSO-*d6*) δ: 51.6 (CH_2_COOH), 42.1 (CH_2_NHOH), 119.2 (CH, C-2’,6’), 124.4 (CH, C-3’,5’), 132.8 (CH, C-3,5), 137.3 (CH, C-2,6), 155.4 (C, C-1), 84.3 (C, C-4’), 157.1 (C, C-4), 160.2 (C, C-1’), 166.1 (C, CONHOH), 175.8 (C, COOH). LC-MS (ESI^+^ ): Found 505.271 (M+H), (calculated for C_16_H_15_IN_2_O_7_S 505.9); 303.4, 243.4, 183.4, 126.9. Elemental analysis (C,H,N) calculated (C 39.10% H 3.10%, N 5.67%) found (C.38.9%, H. 3.09%, N. 5.66%).

*([4-(4-Bromo-phenoxy)-benzenesulphonyl]-hydroxycarbamoylmethyl-amino) acetic acid* *(****10d****)*

Compound **10d** was prepared following the procedure described for **10a** with reagent quantities adapted to the amount of starting material **9d** (1.90 g, 4.30 mmol). Yield: 1.80g, 3.90 mmol, 91 %, white solid. *R_f_* 0.37 (hexane:ethylacetate 4:1).

^1^H-NMR (DMSO-*d6*) δ: 7.74 (d, ^3^*J*_HH_ = 8.8 Hz, 2H, *H*-2,6), 7.50 (d, ^3^*J*_HH_ = 8.8 Hz, 2H, *H*-3,5), 6.70-6.55 (m, 4H, *H*-2’,3’,5’,6’), 4.02 (s, 4H, C*H*_2_), 3.91 (s, 4H, C*H*_2_). ^13^C-NMR (DMSO-*d6*) δ: 52.2 (CH_2_COOH), 43.1 (CH_2_NHOH), 117.4 (CH, C-2’,6’), 131.4 (CH, C-3’,5’), 118.7 (CH, C-3,5), 124.8 (CH, C-2,6), 156.0 (C, C-1), 132.8 (C, C-4’), 157.1 (C, C-4), 160.4 (C, C-1’), 166.0 (C, CONHOH), 174.5 (C, COOH). LC-MS (ESI^+^ ): Found 457.984 and 459.976 (M+H), (calculated for C_16_H_15_BrN_2_O_7_S 456.9 and 459.9 and 460.1); 378.5, 318.4, 258.3. Elemental analysis (C,H,N) calculated (C 41.8% H 3.29%, N 6.10%)found (C.42.0%, H. 3.30%, N.6.11 %).

*(Hydroxycarbamoylmethyl-[4-(4-tributylstannanyl-phenoxy)-benzenesulfonyl]-amino) acetic acid (****11****)*

Compound **10c** (0.700 g, 1.11 mmol) was added to a solution of dry toluene (10 mL) and tetrakistriphenylphoshine (0.08 g, 0.06 mmol) under an argon atmosphere and stirred at reflux for 24 hours. The solvent was evaporated and the product was obtained after flash column chromatography (hexane:ethylacetate 4:1). Yield: 0.534 g, 0.801 mmol, 72 %, colourless oil. *R_f_* 0.31 (hexane: ethylacetate 3:1).

^1^H-NMR (DMSO-*d6*) δ: 7.80 (d, ^3^*J*_HH_ = 8.0 Hz, 2H, *H*-2,6), 7.19 (d, ^3^*J*_HH_ = 8.0 Hz, 2H, *H*-3,5), 7.10 (d, ^3^*J* = 8.2 Hz, 2H, *H*-3’,5’), 6.94 (d, ^3^*J*_HH_ = 8.2 Hz, 2H, *H*-2’,6’), 4.04 (s, 2H, C*H*_2_), 3.98 (s, 2H, C*H*_2_), 1.09 (s, 6H, C*H*_2_), 1.00(s, 6H, C*H*_2_), 0.89 (s, 9 H, C*H*_3_), 0.57 (s, 6H, C*H*_2_). ^13^C-NMR (DMSO-*d6*) δ: 11.8 (CH_2_), 13.9 (CH_3_), 29.9 (CH_3_), 28.2 (CH_2_), 43.2 (CH_2_NHOH), 50.1 (CH_2_COOH), 118.1 (CH, C-2’,6’), 125.3 (CH, C-2,6), 127.1 (CH, C-3,5), 131.7 (C, C-1), 135.3 (C, C-4’), 155.4 (C, C-1’), 137.1 (CH, C-3’,5’), 160.0 (C, C-4), 169.0 (C, C, CONHOH),179.0 (C, COOH).

*([4-(4-tributylstannylphenoxy)benzenesulphonyl]-tert butoxycarbonylmethylamino) acetic acid tert-butyl ester* ***(12)***

A stirred solution of hexabutylditin (230 mg, 0.401 mmol), **9d** (100 mg, 0.163 mmol) and Pd(PPh_3_)_4_ (11 mg, 9.01 μmol) in toluene (4 mL) was degassed by bubbling argon through the solution for 2 hours. The reaction mixture was refluxed overnight under argon atmosphere. The solvent was removed by evaporation and the residue was purified by flash column chromatography (eluent: hexane:ethylacetate 4:1). Yield 201 mg, 0.261 mmol, 65 %, colourless oil. *R_f_* 0.35 (hexane:ethylacetate 4:1).

^1^H-NMR (DMSO-*d6*) δ: 7.89 (d, ^3^*J*_HH_ = 8.4 Hz, 2H, *H*-2,6), 7.28 (d, ^3^*J*_HH_ = 8.4 Hz, 2H, *H*-3,5), 7.10 (d, ^3^*J*_HH_ = 9.1 Hz, 2H, *H*-3’,5’), 6.90 (d, ^3^*J*_HH_ = 9.1 Hz, 2H, *H*-2’,6’), 4.10 (s, 4H, C*H*_2_), 1.45 (s, 18H, C*H*_3_), 1.42 (s, 6H, C*H*_2_), 1.27 (s, 6H, C*H*_2_), 1.17 (s, 6H, C*H*_2_), 0.89 (s, 9 H, C*H*_3_). ^13^C-NMR (DMSO-*d6*) δ: 12.7 (CH_2_), 14.2 (CH_3_), 29.0 (CH_3_), 28.4 (CH_2_), 30.2 (CH_2_), 48.7 (CH_2_), 75.1 (C(CH_3_)_3_), 117.4 (CH, C-3,5), 119.4 (CH, C-2’,6’), 124.9 (CH, C-2,6), 132.3 (C, C-1), 134.1 (C, C-4’), 137.3 (CH, C-3’,5’), 155.9 (C, C-4), 160.2 (C, C-1’), 171.4 (C, COO). LC-MS (ESI^+^ ): Found 767.617 (M+H), (calc. for C_36_H_57_INO_7_SSn, 766.621); 367.145, 304.272, 244.247, 167.243. Elemental analysis (C,H,N) calculated (C, 56.40; H, 7.49; N, 1.83) found (C.56.46 H. 7.49%, N.1.84%).

*([4-(4-[^123^I] Iodophenoxy)-benzenesulphonyl]-hydroxycarbamoylmethyl-amino)-acetic acid ([^123^I]****10c****)* *via the electrophilic aromatic substitution reaction*

To a solution of **12** (0.11 mg, 0.14 μmol) in acetic acid (300 μL) was added sodium[^123^I]iodide (2.4 GBq) in a 0.1 M NaOH solution (ca.10 μL), followed by addition of 30% H_2_O_2_ (70 μL). The reaction mixture was vigorously stirred for 5 minutes and reacted for an additional 15 minutes at room temperature. Subsequently the reaction mixture was quenched using a saturated solution of sodium bisulphite (0.1 mL), followed by addition of saturated sodium bicarbonate solution (0.2 mL). A Sep-Pak^®^ C_18_-cartridge was preconditioned using 5 mL of EtOH followed by 10 mL of H_2_O. The reaction mixture was loaded onto the preconditioned Sep-Pak^®^ C_18_-cartridge. The cartridge was washed with water (3 × 10 mL) and dried with a flow of N_2_ to give [^123^I]**8c.** Removal of the *t*-butyl protecting groups was achieved by eluting the C_18_ cartridge with 2 M HCl in diethyl ether (0.5 mL) and the eluate was left at room temperature for 30 minutes and resulted [^123^I]**9c**. The diethyl ether was evaporated under a continuous flow of argon. Dry THF, NMM, and ECF (500 μL, 250 μL, 250 μL) was added and the reaction mixture was cooled to 0°C and left at this temperature for 5 min. The NH_2_OH was freshly prepared by stirring the NH_2_OH x HCL salt with KOH on an ice bath, in methanol, for 15 minutes solution. The THF solution was added drop wise to the freshly prepared solution of NH_2_OH while being stirred and subsequently left for 15 min at 0°C. The reaction mixture (2 mL) was loaded onto a preconditioned Sep-Pak^®^ C_18_-cartridge and washed (3 × 10 mL H_2_O). The crude product was eluted with EtOH from the Sep-Pak^®^ (500 μL). The ethanol solution, containing the product [^123^I]**10c**, was subjected to high performance liquid chromatography (HPLC) purification on a Cromasil C18 column with 25% acetonitrile (can) / 0.08M Phosphate buffer in H_2_O as eluent at a flow of 5 mL/min. Radio chemical yield was 4 % (decay corrected) and the (radio)chemical purity was > 98%, specific activity 85%.

*([4-(4-[^123^I]Iodophenoxy)benzenesulphonyl]hydroxycarbamoylmethylamino) acetic acid ([^123^I]****10c****) via the nucleophilic aromatic substitution reaction*

To a conical V-vial was added 2,5-dihydroxybenzoic acid (5.0 mg, 32 µmol), citric acid monohydrate (8.0 mg, 38 µmol) and SnSO_4_ (0.2 mg, 0.9 µmol) and 0.2 mL of ethanol containing 1.5 mg (3.3 µmol) of **10d**. Subsequently, 30 μL of a 15 mM copper(II)-sulphate solution and sodium [^123^I]iodide (2.6 GBq) were added. The mixture was freed from oxygen via gentle bubbling the solution with N_2_ for 20 minutes at room temperature. Next, the mixture was heated at 140°C for 45 minutes. After cooling to room temperature the mixture was diluted with 1.0 mL of 10% methanol solution in water, filtered over a 0.45 μm Millex-GV filter and purified using a semi preparative HPLC system. The collected HPLC fractions were diluted with 1.5 times the collected volume with water and the resulting solution was passed through a C18 Sep-Pak Classic. Next the cartridge was rinsed with 10 mL of sodium hydroxide solution (pH 9) and subsequently with 10 mL of water. The product was recovered from the cartridge with 1.0 mL of a 0.6% solution of sulphuric acid in ethanol. Overall yield was > 51% with a (radio) chemical purity of >99% and a specific activity of > 80 TBq/μmol.
