# Supplementary figures and images for: Novel molecular imaging ligands targeting matrix metalloproteinases 2 and 9 for imaging of unstable atherosclerotic plaques
Source: PLoS One. 2017 Nov 30;12(11):e0187767. doi: 10.1371/journal.pone.0187767 (PMC5708805; doi:10.1371/journal.pone.0187767)

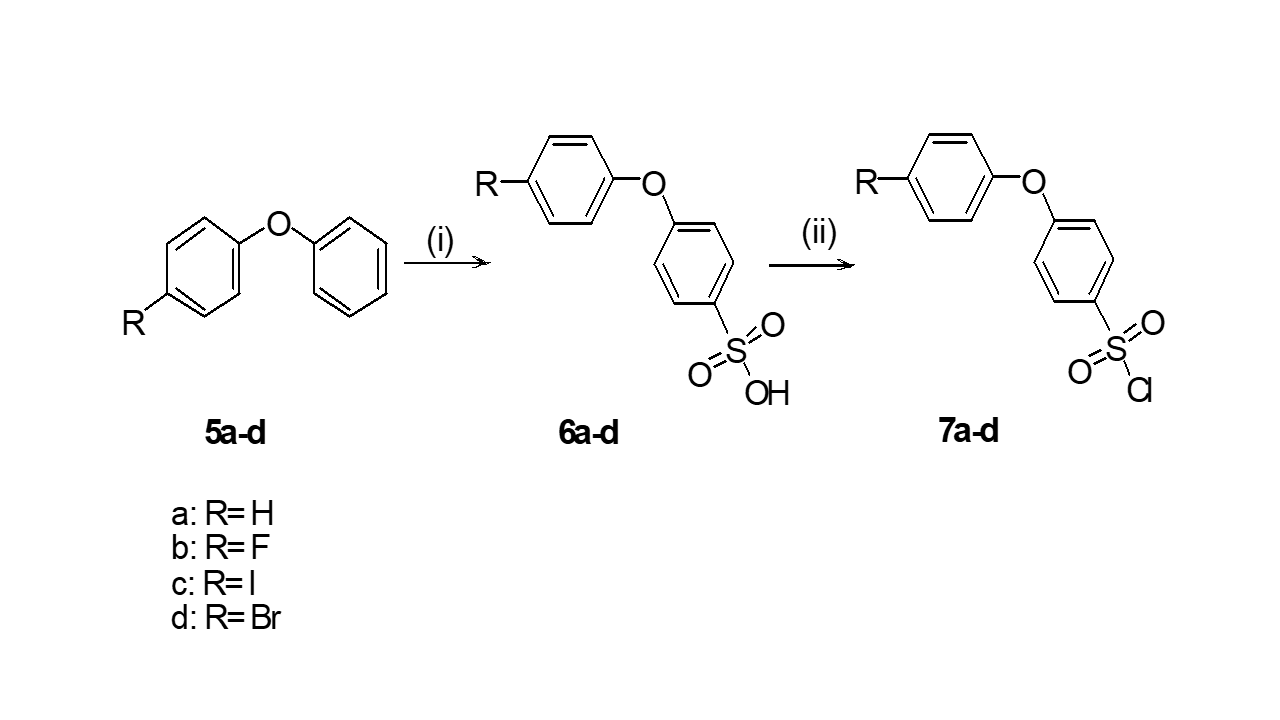

Supplement: S1 Fig — Synthesis of the arylsulfonylchlorides 7a-d from 5a-d (i): ClSO3H, CH2Cl2, 0°C, 2h, (ii): SOCl2, DMF (cat), reflux, 6h, yield 80–97%. (TIF) [file pone.0187767.s003.tif]

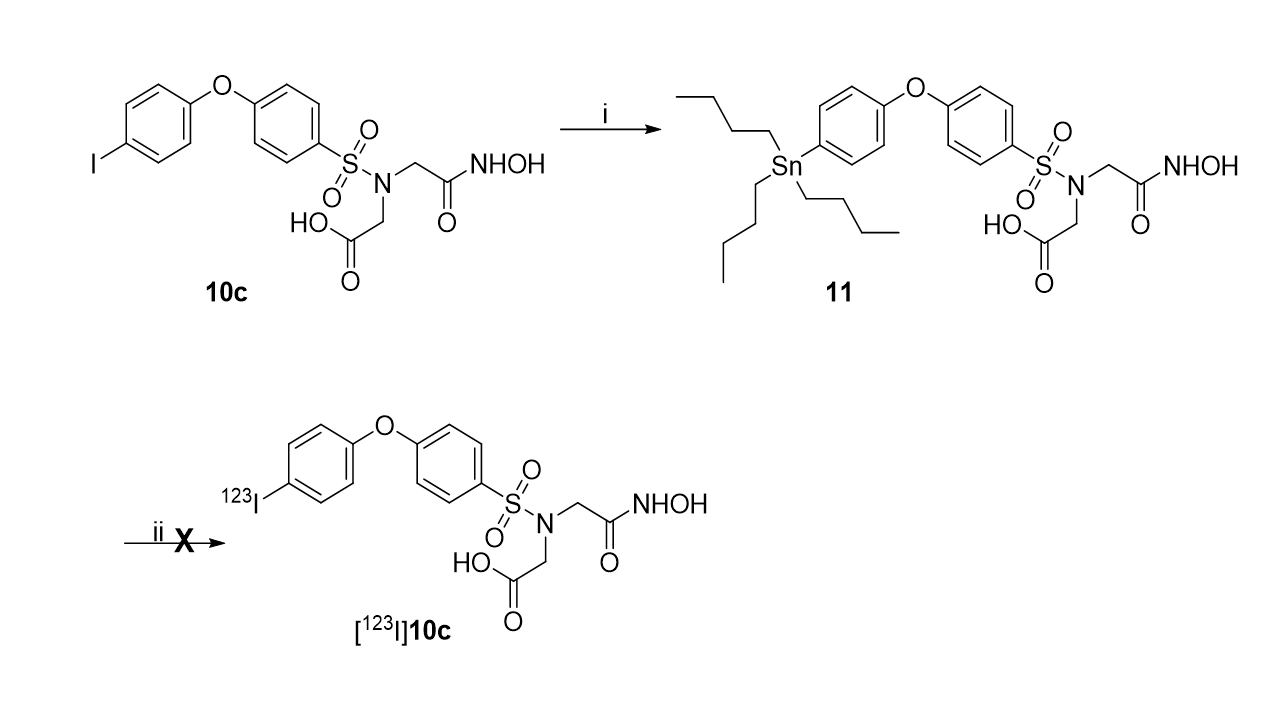

Supplement: S2 Fig — Attempted radiosynthesis of [123I]10c (i): (n-Bu3Sn)2, Pd(PPh3)4, toluene, reflux, o/n, yield 72%; (ii): Na[123]I, H2O2, CH3COOH, rt, 20 min, yield 0%. (TIF) [file pone.0187767.s004.tif]

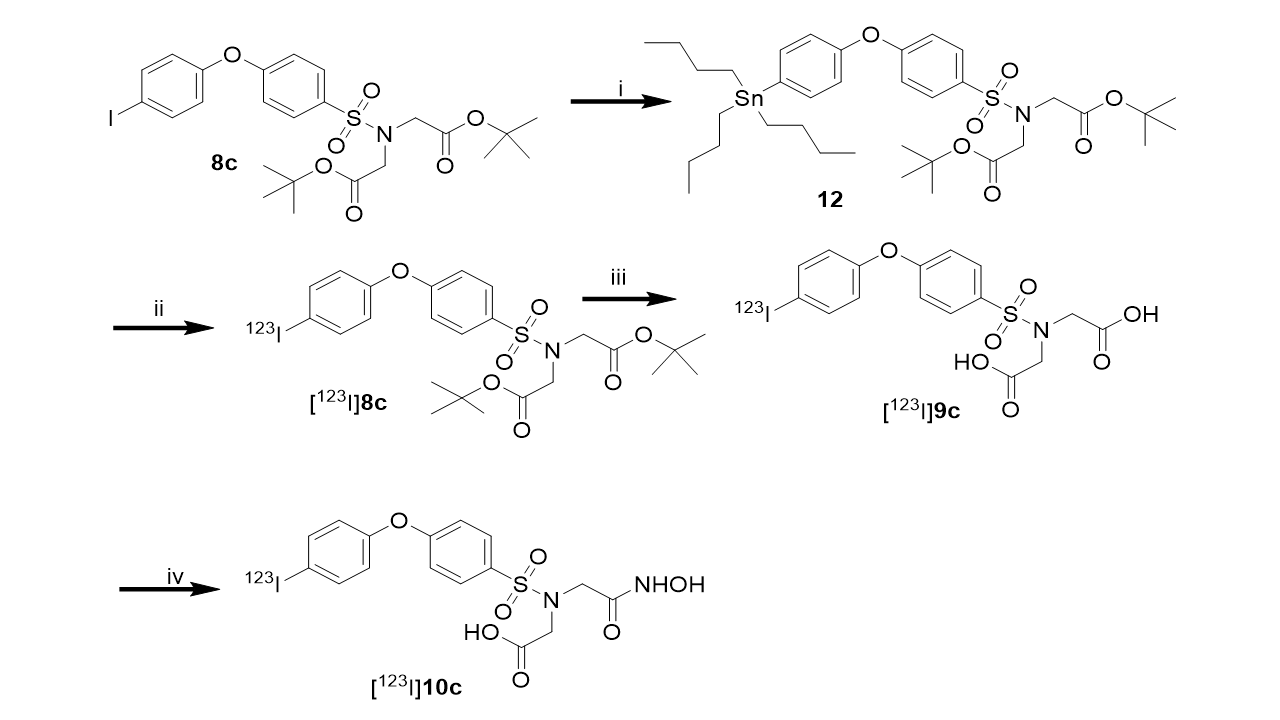

Supplement: S3 Fig — Radiosynthesis of [123I]10c from 8c via electrophilic radioiodination. (i): (n-Bu3Sn)2, Pd(PPh3)4, toluene, reflux, o/n, yield 76%; (ii): NaI[123], H2O2, CH3COOH, 25 min.; (iii): 2M HCl in Et2O, rt, 30 min.; (iv): a) ethylchloroformate, NMM, THF, 15 min, b) NH2OH x HCl, MeOH, 0°C, 15 min. Overall (steps ii to iv) decay corrected radiochemical yield of [123I]10c was 4%. (TIF) [file pone.0187767.s005.tif]
